# AccES: A Framework for Predicting Building‐Level Loss of Accessibility to Essential Services Post‐Disasters Based on Publicly Available Information

**DOI:** 10.1111/risa.70373

**Published:** 2026-10-07

**Authors:** Zaira Pagan‐Cajigas, Seth Guikema

**Affiliations:** ^1^ Industrial & Operations Engineering University of Michigan Ann Arbor Michigan USA; ^2^ Civil & Environmental Engineering University of Michigan Ann Arbor Michigan USA

**Keywords:** accessibility, disaster resilience, equitable access, essential services, infrastructure interdependencies

## Abstract

Natural hazards significantly disrupt access to essential services, affecting individuals' capacity to withstand and recover from such events. However, existing hazard resilience models often fail to account for the interdependencies among essential services and lack the spatial resolution needed to assess accessibility loss at the building level. This study introduces the **Predicting Loss of Access to Essential Services (AccES)** framework—a novel, data‐driven, and people‐centered approach that uses publicly available information to evaluate accessibility following a natural hazard event. The framework integrates open‐source datasets with local stakeholder knowledge to develop spatially explicit models of interconnected infrastructure networks, including power, water, and communication networks, along with their dependent service facilities such as hospitals, childcare centers, and grocery stores. Accessibility loss is assessed by evaluating the disruptions to infrastructure networks and service facilities, estimating (1) the probability that individual buildings lose access to infrastructure services and (2) the expected increase in travel distance to the nearest operational service facility. To demonstrate the framework's utility, a case study was conducted in Cayey, Puerto Rico, simulating the impacts of a Category 2 tropical cyclone. Results highlight spatial disparities in accessibility loss, with rural and socially vulnerable areas facing greater burdens. AccES advances hazard resilience modeling by providing a scalable, equity‐oriented tool to support data‐informed decision‐making for both pre‐disaster planning and post‐disaster response.

## 1 Introduction

Over the past two decades, more than 1.5 billion people have been affected by natural hazards, resulting in over 700,000 deaths and $1.3 trillion in economic losses (UNDRR and WMO 2022). Natural hazards cause significant disruptions to essential services, including both infrastructure networks and the service facilities they support (United Nations Office for Disaster Risk Reduction [UNDRR] 2015; Tierney 2019). These services are highly interconnected, meaning disruptions in one can propagate into failures across multiple services, impacting community safety, health, and overall well‐being (E. A. Singh 2020).

Traditional engineering disaster risk and resilience models primarily focus on physical infrastructure damage and recovery, implicitly assuming uniform consequences across the affected population. However, this approach overlooks the disproportionate impact of service disruptions on vulnerable groups and does not directly consider loss of non‐infrastructure but still essential services such as grocery stores, healthcare, and childcare. For example, after Hurricane Maria impacted Puerto Rico, the failure of the power grid triggered cascading effects across dependent infrastructure networks, including water, communication, and transportation networks, which in turn disrupted essential service facilities such as hospitals, grocery stores, and gas stations (Chandra et al. 2021). As a result, residents had to travel significantly longer distances to access the limited medical care, food, and fuel resources. However, this burden was not equally distributed. Research shows rural, low‐income residents were disproportionately affected due to pre‐existing disparities in financial resources, mobility, and infrastructure resilience, inequities that were further exacerbated after the disaster (Szczyrba et al. 2021).

Access to essential services is critical for recovery, enabling individuals to regain a sense of normalcy after a disaster. When these services are unavailable or inaccessible, communities face prolonged instability, often forcing residents to relocate or causing long‐term socio‐economic consequences (Contreras et al. 2017). As climate change continues to intensify the frequency and intensity of weather‐related disasters (Intergovernmental Panel on Climate Change [IPCC] 2023), risk and resilience models must evolve to not only account for infrastructure disruptions but also to assess the resulting loss of access to services. This requires data‐driven assessments at fine spatial scales that integrate equity considerations and identify population‐specific vulnerabilities.

In this paper, we introduce the Predicting Loss of Access to Essential Services (AccES) framework, a novel, data‐driven, and people‐centered tool designed to evaluate accessibility loss at a building level following a hazard event. AccES represents a significant advancement in disaster resilience modeling by using publicly available datasets to develop spatially explicit representations of an interconnected essential services network. The model quantifies loss of accessibility across services such as power, water, and communication networks and the service facilities they support, such as hospitals, grocery stores, childcare, and eldercare facilities.

A key feature of AccES is its incorporation of multi‐sectoral local knowledge through stakeholders’ interviews, governmental agency inputs, and community engagement, enabling the tool to reflect the unique needs and priorities of the community. While the framework is hazard‐agnostic, we demonstrate its application in the context of a tropical cyclone. Our case study simulates service accessibility loss in Cayey, Puerto Rico, a municipality highly susceptible to natural hazards.

The remainder of this paper is structured as follows: Section 2 provides an overview of existing approaches for measuring post‐hazard impact at the community level. Section 3 outlines the methodology of the AccES framework. Section 4 presents a case study in Cayey, Puerto Rico. Section 5 concludes with a discussion of contributions, practical implications, and future research directions.

## 2 Background

Quantifying resilience at the local level is critical for informing targeted mitigation, preparedness, response, and recovery interventions, particularly in hazard‐prone regions. However, there is no universally accepted method for measuring resilience, and existing approaches vary in focus and scope (Johansen et al. 2016; Patel et al. 2017; Nguyen and Akerkar 2020). The two dominant approaches are (1) community‐level adaptive capacity models (Cutter et al. 2014; Food and Agriculture Organization of the United Nations [FAO] 2016; Gillespie‐Marthaler et al. 2019; Tariq et al. 2021), and (2) engineering‐based models (Klise et al. 2017; Mottahedi et al. 2021). These existing models range from highly localized component‐level to broader national‐level resilience metrics.

The community's adaptive capacity is often estimated using aggregated indicators across socio‐demographic, economic, infrastructure, environmental, and institutional dimensions (Norris et al. 2008; Cutter et al. 2010; Sherrieb et al. 2010; Summers et al. 2018; Feldmeyer et al. 2021; Edgemon et al. 2023; Camacho et al. 2023). These estimations are typically reported as an aggregated metric in the form of scorecards, indices, and toolkits at the census tract or county‐level. By providing only an aggregated metric for the study area, these results often overlook resilience disparities at finer spatial scales. In addition, many of these models have been validated for a single case study, which limits the applicability of their identified indicators to other communities (Burton 2014). Moreover, most do not account for hazard‐specific dynamics, thereby failing to capture how resilience may vary depending on the type or intensity of disruption.

Traditional engineering‐based models, on the other hand, predominantly focus on physical infrastructure performance (Klise et al. 2017; Guikema and Gardoni 2009; Righi et al. 2015; Mottahedi et al. 2021). These models primarily evaluate infrastructure damage and recovery at the asset level, often using fragility and recovery curves. These assessments provide quantifiable insights into the system's performance, both structurally and functionally. However, focusing only on the physical infrastructure fails to account for disparities in household‐level consequences, which vary depending on an individual's resources and capabilities (Tierney 2019).

Moreover, many engineering‐based models focus on a single infrastructure network, failing to capture the cascading effects across interdependent services. Recognizing this limitation, recent research has emphasized the need to account for interdependencies when evaluating infrastructure network resiliency (Cimellaro et al. 2024; Guidotti et al. 2016; Fekete 2020). Some studies, such as Brunner et al. (2023), have advanced this field by estimating disruptions across multiple infrastructure networks at the household level. However, such approaches often overlook service facility disruptions, which are critical for post‐disaster recovery. In addition, these models rely on proprietary utility data or use connectivity as an approximation to actual engineering system performance, limiting their ability to perform multi‐network impact assessments to only areas where such data are available and accessible. However, in many regions, especially those with limited or restricted data‐sharing policies, access to utility‐supplied data is unavailable.

A more recent approach by Logan and Guikema (2020) evaluates community resilience by measuring building‐level access to essential services. Access is a multidimensional concept that includes accessibility, availability, acceptability, affordability, adequacy, and awareness (Penchansky and Thomas 1981; Saurman 2015). Their model uses proximity, measured in terms of distance and travel time, as a proxy for access. However, recent research by Enderami et al. (2024) emphasizes the need for complementary metrics that account for the additional dimensions of access, such as the doctor‐to‐patient ratio, to reflect acceptability.

While Logan and Guikema (2020) model provides a strong foundation, its approach is inherently retrospective and limited to post‐disaster evaluations. This limitation is particularly significant in the context of a changing climate, where the ability to model unforeseen disaster scenarios is necessary to anticipate and mitigate future disruptions. In addition, although their model considers access to essential service facilities, it does not account for the infrastructure networks, such as power, water, or communications, required to sustain these services.

To our knowledge, no existing framework both uses publicly available data to model interconnected essential services and quantifies building‐level accessibility loss across multiple services. To address these gaps, we developed the AccES framework, a data‐driven, scalable model that uses publicly available data to simulate access loss at the building level. Unlike prior models, AccES integrates infrastructure network interdependencies and service facility disruptions, allowing stakeholders to assess how different sectors of the community may experience access loss to essential services after a hazardous event, even in the absence of proprietary infrastructure data. However, it is worth acknowledging that the availability of comparable public datasets varies across countries and regions, which may affect the direct applicability of the framework in settings where such data are not available.

## 3 AccES Framework

The AccES framework integrates five interconnected models to estimate building‐level accessibility loss to essential services following a hazard. It incorporates both infrastructure networks and the service facilities they support, while integrating insights from local stakeholders to ensure practical relevance. The methodology consists of two main phases: pre‐hazard assessment and post‐hazard impact analysis, as shown in Figure 1.

**FIGURE 1:**
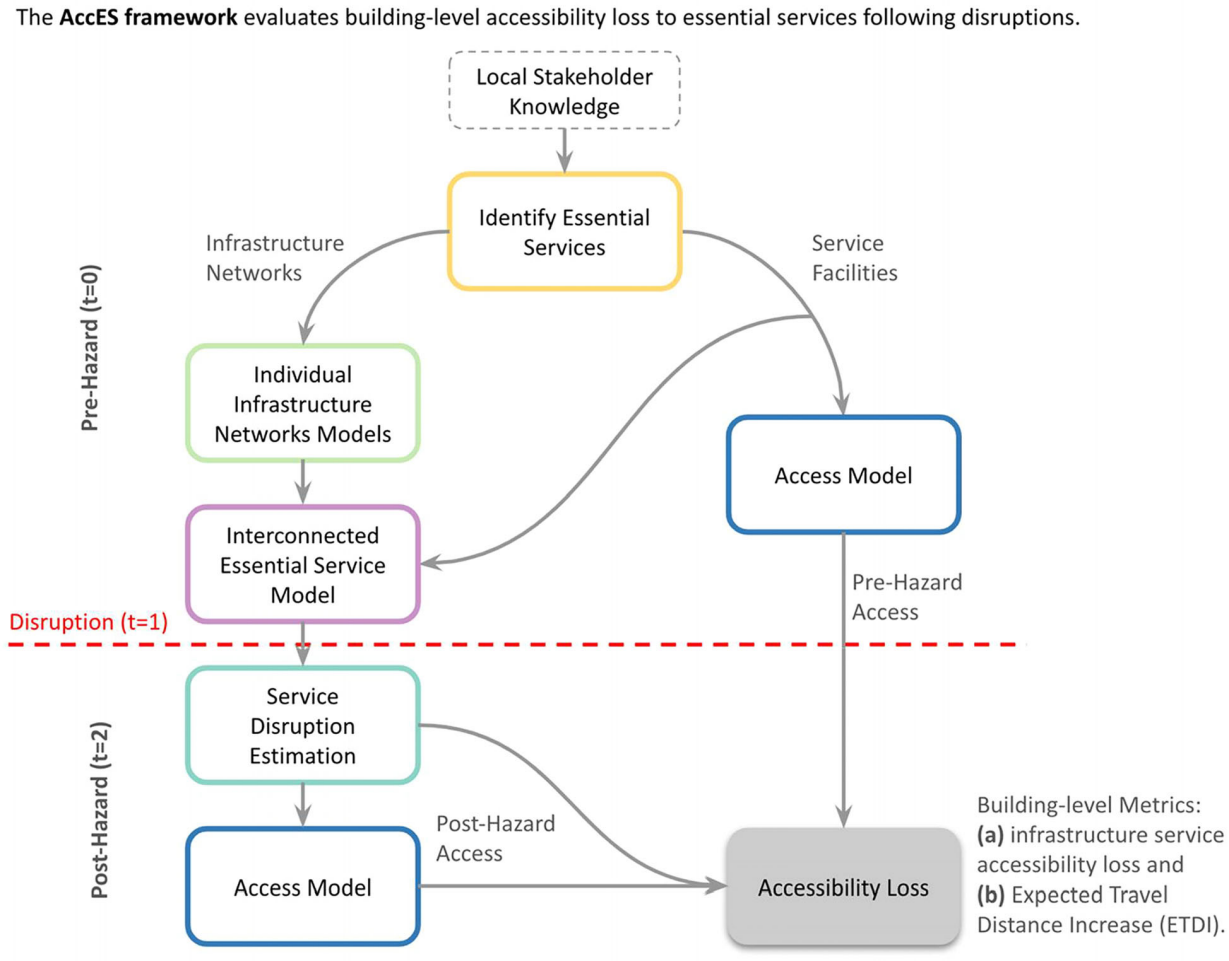
AccES framework flowchart.

In the pre‐hazard assessment, baseline conditions are established by first defining essential services through local knowledge. Infrastructure networks are modeled through spatially explicit, detailed engineering representations of networks using only publicly available data. Dependencies between essential services, both infrastructure networks and service facilities, are incorporated to capture potential cascading failures. Finally, pre‐hazard accessibility is quantified by calculating the distance from each building to the nearest operational service facility, assuming all service facilities are fully operational before the event.

The post‐hazard impact analysis simulates infrastructure disruptions based on potential hazard scenarios and evaluates accessibility loss at the building level. The operational status of service facilities is determined based on their dependencies and access to infrastructure network services. Accessibility loss is measured by (a) the loss of infrastructure network services, and (b) the increase in travel distance to the nearest operational essential service facility compared to pre‐hazard conditions.

### 3.1 Pre‐Hazard Assessment

#### 3.1.1 Local Stakeholder Knowledge

Defining what constitutes “essential” is inherently community‐dependent, influenced by the community's societal context, including cultural norms, sociodemographic, and socioeconomic conditions (Logan and Guikema 2020). Integrating local knowledge ensures that essential services are identified based on the specific needs of the community. Relying only on a predefined national‐level list of essential services may overlook local needs (Federal Emergency Management Agency [FEMA] 2011). For example, while childcare services may not be recognized as essential at a national level (Homeland Security Presidential Directive 2003; Norwegian Directorate for Civil Protection 2017, FEMA 2023), the lack of childcare can significantly impact parents', particularly single parents, ability to work, support their families, and contribute to the local economy.

To ensure the AccES framework reflects the local priorities, we conducted in‐depth, semi‐structured interviews with stakeholders from both rural and urban areas of the case study community. These local inputs shaped multiple components of the AccES framework. Particularly, on defining essential services, informing service dependencies across infrastructure and facilities, and identifying vulnerable populations for equity‐centered analysis.

These stakeholders included community leaders, essential service personnel, and emergency management officials. As our case study was Cayey, PR, all interviews were conducted in Spanish to ensure participants could express themselves comfortably and naturally. All recordings were subsequently translated and transcribed into English for analysis.

Eleven interviews were conducted with community leaders (e.g., civic, religious, political), identified by local government as representatives of diverse sectors. To ensure relevant experience, participants were required to be permanent residents of Puerto Rico, at least 18 years old, and have a minimum of five years of community engagement as of 2021. This ensured that all interviewees had lived through and responded to at least one major hazard event.

The interviews provided critical insights into locally relevant essential services, including shelter, water, energy, transportation, communications, food supply, healthcare, education, childcare, eldercare, security, civic organizations, congregations, and financial services. Notably, several of these services, such as childcare and congregations, are not listed in national‐level classifications of critical infrastructure, including FEMA's Community Lifelines. This highlights the importance of incorporating local knowledge, as overlooking community‐specific priorities can lead to gaps in both mitigation planning and post‐disaster restoration. Importantly, community leaders also highlighted the needs of vulnerable populations, particularly rural, elderly, and low‐income residents.

Interviews with technical and facility‐level stakeholders from the water utility personnel provided technical validation into the synthetic network model and provided insights into the system dependencies and power backup capacity. Shorter interviews with six service facility managers (e.g., schools, grocery stores, childcare centers) provided data on facility‐level service continuity strategies and operational capacity during disruptions. In addition, interviews with two emergency management officials provided insights on restoration priorities and interdependencies based on prior disaster response experience.

#### 3.1.2 Individual Infrastructure Network Models

To model infrastructure networks at a fine spatial scale, this study uses synthetic, spatially explicit engineering models built from publicly available data. This approach addresses the common challenge of limited access to detailed infrastructure information, often restricted for security or proprietary reasons. Synthetic modeling offers a practical alternative to estimating hazard impact, particularly in communities where high‐resolution infrastructure data is unavailable.

This work builds on previously developed models (Zhai et al. 2020; Pagan‐Cajigas et al. 2025, Feeny et al. 2026) to develop individual synthetic networks for power, water, and communication systems. The power distribution model, based on Zhai et al. (2020), generates a connectivity network using roads, substations, and building data. The water distribution model, based on Pagan‐Cajigas et al. (2025), simulated the hydraulic performance at the building‐level using roads, water treatment plants (WTP), tanks, and building data. The communication model, based on Feeny et al. (2026), utilized cell tower and antenna data to estimate cellular coverage.

##### 3.1.2.1 Power Distribution Model

The synthetic power distribution network was developed using the model of Zhai et al. (2020), which generates power network layouts for any US region using only publicly available data. This model has been validated against real US power systems. It incorporates road networks, substation locations, and building footprints to generate a node‐edge power distribution network, which accounts for substations, poles, and distribution lines. Road data was obtained from the U.S. Census Bureau (2021), while substation locations and building footprints were obtained from OpenStreetMap (2017).

This network is developed on the following assumptions: (1) power lines are located within roads' right‐of‐way, (2) the network follows a least‐cost design, and (3) the network operates as a radial system, where each building is connected to the nearest substation using the weighted shortest path algorithm. Utility poles are then placed along the shortest path route, with a predefined spacing of 40 meters to approximate real‐world conditions. In our synthetic system, all poles are assumed to be overhead, reflecting the existing infrastructure in the case study community. However, the original model of Zhai et al. (2020) included underground distribution circuits for other areas as appropriate.

Once poles are placed, distribution lines are integrated into the model, establishing connections between customers and substations. Given the network's radial structure, failures at any pole or distribution line disrupt all downstream components. In other words, if a pole is damaged, all connected poles and distribution lines beyond that point become non‐operational. This simulates real‐world cascading outages, where a single‐point failure can lead to widespread service loss.

It is important to note that this synthetic model represents only the network's connectivity and does not simulate power flow dynamics or load balancing. Further details on the methodology and validation of the synthetic power system are provided by Zhai et al. (2020).

##### 3.1.2.2 Water Distribution Model

The synthetic water distribution system (WDS) builds on the methodology by Pagan‐Cajigas et al. (2025), which uses publicly available data to generate a spatially explicit network layout and simulate hydraulic behavior. The WDS layout is modeled as a node‐edge network constructed by integrating road networks, building footprints, and the locations of above‐ground infrastructure components such as WTP and storage tanks, obtained from sources such as the U.S. Census Bureau, OpenStreetMap (OSM), and local data where available. Roads (excluding highways and ramps) are used as a proxy for pipe placement, while buildings are used to estimate spatially distributed demand.

The building classification, obtained from the building footprint, is used to assign daily water demand patterns, which are allocated to the nearest node to ensure an accurate spatial distribution of demand across the system. Pipe length and diameter are based on road classification, while pipe material and roughness coefficient are specified using a predefined pipe dictionary included in the model. Storage tank capacities are initially allocated based on an equal distribution of the system's total water demand. Tank dimensions are calculated using standard industry sizing guidelines for double ellipsoid and elevated toro tanks. Pumps are assumed to be located between the main pipes and the storage tanks, reflecting a simplified but practical system configuration. However, when more detailed information on pump locations and capacities is available, it can be integrated into the model to enhance accuracy. The WTP(s) are modeled with the assumption of an infinite water supply; however, this assumption can be modified to reflect more restrictive water supply conditions.

Hydraulic simulations are conducted using the WNTR package in Python. An initial demand‐driven simulation established baseline pressure conditions, followed by k‐means clustering to define pressure zones. Tank and pump parameters are then updated to reflect the demand requirements within each zone, then a second demand‐driven simulation is performed to ensure all buildings meet the minimum service pressure (≥ 20 PSI).

In pressure‐deficient scenarios, the water distribution model switches to a pressure‐driven simulation to account for system failures, such as pump disruptions caused by power outages or pipe breaks. Nodes with pressure below 20 PSI are classified as failed, indicating insufficient service levels. This approach allows the model to assess not just network connectivity but also the ability to maintain adequate pressure under disrupted conditions. Further details on the methodology and validation of the synthetic WDS are provided in Pagan‐Cajigas et al. (2025).

In our case study of Cayey, Puerto Rico, we had access to detailed infrastructure data (Autoridad de Acueductos y Alcantarillados [AAA] 2015), which enabled model refinement. This included precise component attributes (e.g., pipe diameter, material, roughness, tank dimensions, WTP capacity, pump flow rates). Given the available data, we enhanced the original model by incorporating pressure‐control valves. This addition is particularly important for simulating adequate pressure regulation in systems with substantial elevation variability. In addition, unlike the original model, which dynamically assigns pressure zones, we utilized pre‐defined pressure zone classifications from the available dataset. Where classifications were missing, we applied a k‐means clustering algorithm to infer zone classification based on spatial proximity to known zones.

In contexts where such detailed datasets are not available, the framework defaults to the original model by Pagan‐Cajigas et al. (2025). This flexibility ensures the model's applicability across a wide range of communities with varying levels of data availability.

##### 3.1.2.3 Communication Model

The communication model was developed using data from OpenCelliD, an open‐source database that provides detailed information on each cellular tower and antenna, including radio type, geographic coordinates, and coverage area. The location and coverage range are derived from aggregated GPS measurements. Previous validation studies have shown that OpenCelliD offers reasonably accurate component location estimates, with a median localization error of approximately 508 meters and a mode near 200 meters (Ulm et al. 2015). However, antennas with estimated coverage radii below 50 meters tend to exhibit unreliable spatial accuracy due to data biases. In this study, all antennas and towers had an estimated coverage radius exceeding 1000 m.

To represent network coverage, we generated a coverage map by assuming a circular coverage radius around each tower and antenna, following the methodology of Feeny et al. (2026). Using this approach, buildings are assigned to all overlapping service areas, capturing the redundancy typical of real‐world cellular networks, especially in urban settings where overlapping coverage improves reliability and service continuity.

During a disruption scenario, the model simulates failures by removing the coverage areas of damaged towers or antennas. After removing all damaged infrastructure, the model evaluates whether each building remains connected to at least one operational tower or antenna. This assessment is limited to coverage area availability and does not account for potential service degradation caused by increased demand on the remaining network infrastructure.

#### 3.1.3 Interconnected Essential Service Model

Essential services are highly interconnected, meaning a single failure can propagate disruptions across multiple systems (A. N. Singh et al. 2014; Ciornei et al. 2017; Palleti et al. 2021). While modeling individual infrastructure networks provides valuable insights, it does not capture the cascading effects of system failures (Brunner et al. 2023). To address this limitation, we modeled dependencies as directional edges linking dependent components (nodes) across different networks.

Dependencies are often classified as physical, cyber, geographical, or logical (Rinaldi et al. 2001). Physical dependencies occur when the system relies on the output of another. Cyber dependencies happen when systems are interconnected through information exchange. Geographical dependencies arise from spatial proximity, increasing the likelihood of simultaneous failures during localized hazard events. Logical dependencies include all other interconnections, including policies or institutional decisions that influence multiple systems.

The interconnected essential service model focuses on physical dependencies, capturing interconnections (a) within infrastructure networks and (b) between infrastructure networks and service facilities. Due to data limitations, cyber, geographical, and logical dependencies are not included in this analysis. However, integrating these dependencies in future model development would provide a better understanding of cascading failures and their broader impact on community resilience.

##### 3.1.3.1 Infrastructure Network Dependencies

Our model captures first‐order physical dependencies amongst infrastructure systems, specifically focusing on the reliance of water and communication networks on the power network, as shown in Figure 2. Research by A. N. Singh et al. (2014) indicates that infrastructure networks can be classified by their levels of dependency and driving power, with the power network considered functionally independent due to its minimal reliance on other networks. In contrast, water and communication networks require a continuous power supply to remain operational (Anderson et al. 2020; National Association of State Energy Officials [NASEO] 2021).

**FIGURE 2:**
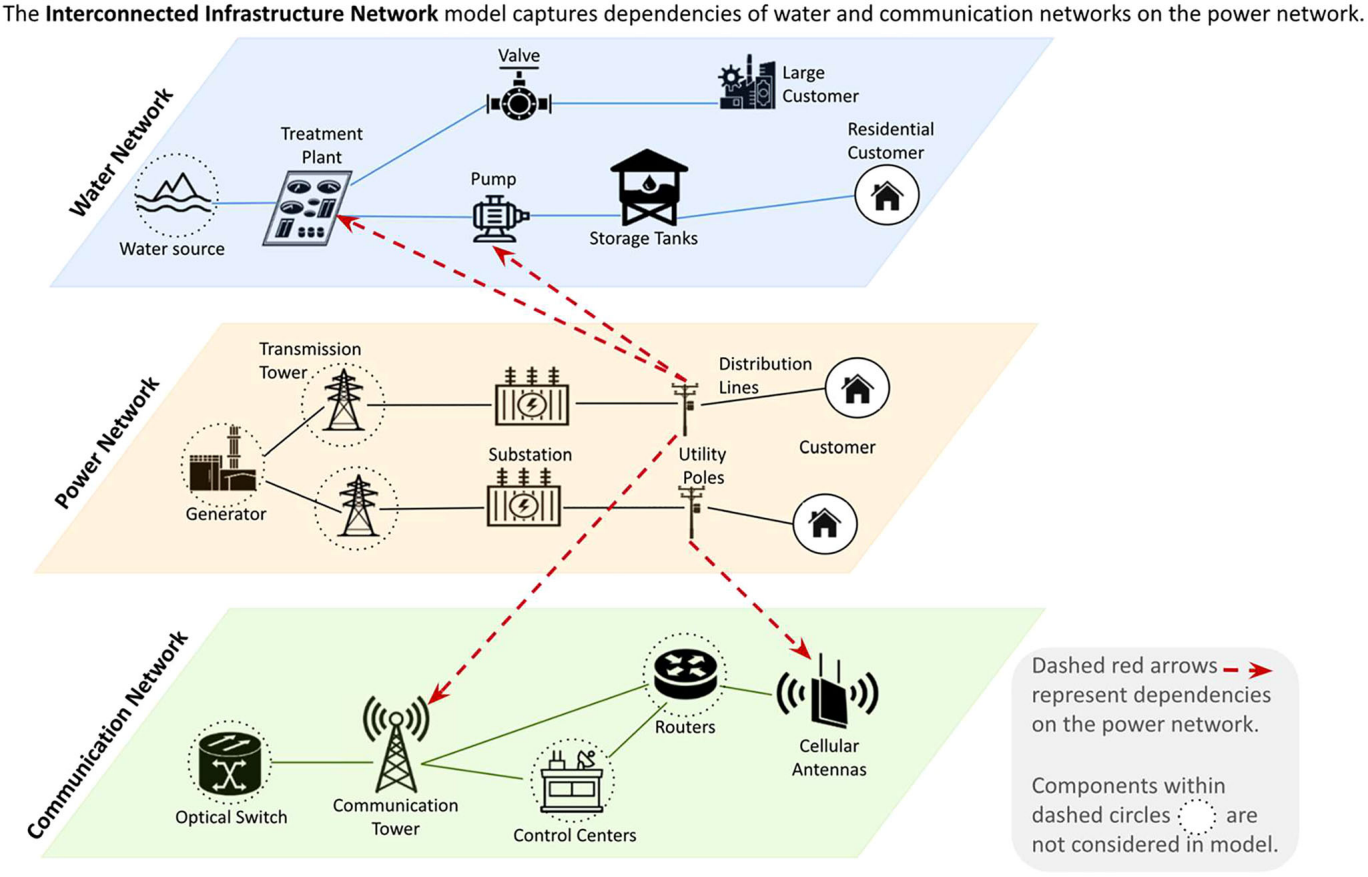
Infrastructure network dependencies.

To represent these dependencies, WTPs, pumps, antennas, and cellular towers are linked to the nearest power distribution pole. Each pole's operational status is modeled as a binary variable: 1 for functional (supplying power) and 0 for non‐functional. This binary status is then used as an input to the water and communication system models, defining the impact on the functionality of dependent components.

Power disruptions significantly impact the WTPs and pump stations, which are essential for maintaining the water supply and quality (Palleti et al. 2021; U.S. Environmental Protection Agency [EPA] 2023). A failure at the WTP can compromise water quality and increase the risk of contamination. Similarly, pump failures can lead to insufficient water pressure, causing service interruptions at the building level and disrupting other essential services, such as firefighting operations (EPA 2023). Given their critical nature, most WTPs are equipped with backup generators to maintain operations during outages (Argonne National Laboratory 2016). Accordingly, our model assumes WTPs remain operational during power failures. However, not all pump stations have alternative power sources. To address this, we integrated utility‐reported data identifying pump stations with alternative power sources. During a power outage (i.e., pole status = 0), dependent pumps without backup generation are assumed to be non‐operational.

Similarly, the functionality of antennas and communication towers is highly dependent on power availability. During outages, communication systems lose functionality unless equipped with alternative power sources (Johansson and Hassel 2010; Feeny et al. 2026). These disruptions can severely impact emergency response coordination and limit access to vital information.

However, detailed data on which specific components have backup systems is limited (U.S. Government Accountability Office [GAO] 2021a, 2021b). Due to these data limitations, we assume that 25% of towers and antennas have backup power, selected based on maximum coverage, prioritizing transmission towers given their typically larger service areas. As more detailed data and industry standards become available, these assumptions can be refined. Future model iterations can integrate such information to enhance the accuracy of resilience assessments.

We acknowledge that there exist interdependencies between the water and communication networks. For example, water is often required for cooling communication infrastructure, and communication networks support the operation of water supply components, such as pumps and storage tanks, through cyber‐based controls. The power system can also depend on both networks, requiring water for cooling in power generation or relying on communication for grid management and coordination (Rinaldi et al. 2001). However, these dependencies are beyond the scope of our analysis.

##### 3.1.3.2 Service Facilities Dependencies

Our model accounts for the physical dependencies of essential service facilities, such as hospitals, schools, childcare centers, and eldercare facilities, on infrastructure networks, particularly power and water. These dependencies were identified through existing research, regulatory guidelines, and direct input from the service personnel. For example, in states like Louisiana, schools and colleges are required by law to maintain adequate water access to remain operational (Louisiana State Legislature 2024).

Childcare centers and schools depend on power and water for heating and cooling systems, sanitation, food preparation, laboratory functions, and general maintenance. Hospitals, veterinary services, and eldercare facilities are highly dependent on electricity for life‐support systems, medical equipment, and electronic health records, while water is essential for sanitation, infection control, and hygiene standards (World Health Organization 2023). Similarly, gas stations require power to operate fuel pumps, and grocery stores depend on continuous electricity for refrigeration and cold storage to prevent food spoilage.

In our model, the operational status of hospitals, veterinary clinics, childcare centers, schools, and eldercare facilities depends on access to both power and water, while gas stations and grocery stores only require power to remain operational, as shown in Figure 3. The model also includes the capacity to integrate alternative power and water sources, such as backup generators and cisterns, at essential service facilities. The initial analysis assumes no backup systems are in place. However, in subsequent scenarios, we incorporate backup power for selected facilities to assess how resilience measures affect service accessibility at the building level.

**FIGURE 3:**
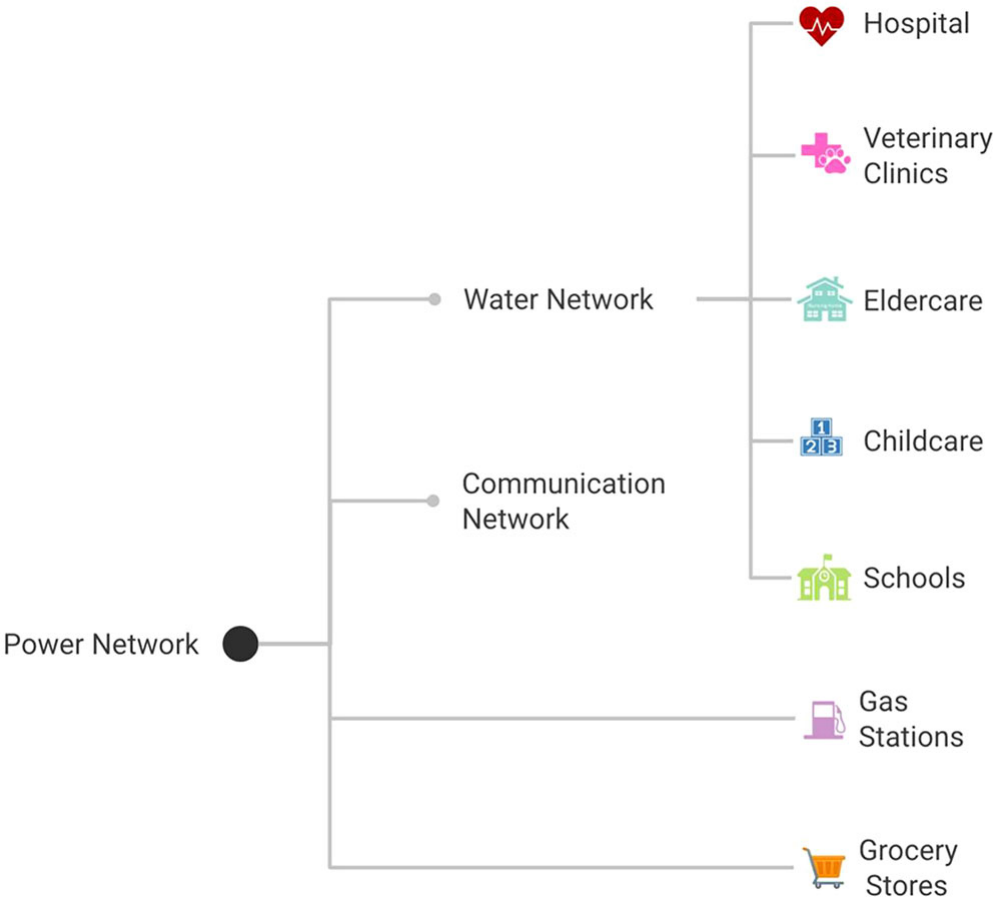
Service facilities dependency on power and water networks.

#### 3.1.4 Access Model

The Access model assesses both network‐based and facility‐based accessibility under pre‐ and post‐hazard conditions by (a) evaluating whether individual buildings have access to infrastructure network services and (b) calculating the road distance to the nearest operational service facilities.

##### 3.1.4.1 Access to Infrastructure Network Services

Building‐level access to infrastructure services is modeled using a binary variable $a_{i,j}$, where i represents the building and j the specific service. Each building is assigned three values $a_{i,1}$, $a_{i,2}$, $a_{i,3}$, indicating access to power (j=1), water (j=2), and communication (j=3). A value of $a_{i,j}$ = 1 indicates access, while $a_{i,j}$ = 0 indicates no access.

Under pre‐hazard conditions, the model assumes uninterrupted access to all three services. In post‐hazard scenarios, power and communication networks are modeled as connectivity‐ and coverage‐based networks, with binary outcomes: service is either available or unavailable. Water access is similarly represented as a binary variable, but it depends both on network connectivity and hydraulic performance. The hydraulic model evaluates pressure levels at each pipe segment, and a building is considered to have access if the connected pipe maintains a pressure of at least 20 PSI. This threshold ensures that the building not only receives water but also has an adequate supply for functional use.

##### 3.1.4.2 Access to Service Facilities

Building‐level access to service facilities is quantified using road network distance to the nearest operational service facility, following the framework proposed by Logan and Guikema (2020). Service facility locations are identified using open‐source datasets, such as OSM, Google, and community‐specific datasets. For each building, road network distances are calculated to all service facilities to identify the nearest operational provider. This method assumes that individuals will seek services based on the shortest distance along existing road networks. Although other factors, such as cost, service quality, or personal preferences, may also influence consumer behavior, these considerations are beyond the scope of this analysis.

To visualize and quantify accessibility, the model generates both accessibility maps and cumulative distribution functions. The accessibility maps depict buildings color‐coded by their proximity to the nearest operational service facility. The cumulative distributions provide a quantitative assessment of the percentage of buildings within varying distance thresholds of essential services facilities.

Under pre‐hazard conditions, all facilities are assumed to be operational. In post‐hazard scenarios, however, service availability is reassessed based on infrastructure dependencies, as detailed in Section 3.1.3.2. For instance, if an eldercare facility does not have power, it is classified as non‐operational. Once the status of each facility is updated, the model recalculates building distances to the nearest operational facility and updates the accessibility maps and cumulative distributions to reflect post‐hazard conditions.

### 3.2 Post‐Hazard Assessment

#### 3.2.1 Service Disruption Model

The service disruption model simulates hazard‐induced damage to the interconnected essential service system (Section 3.1.3) and estimates post‐hazard service availability. The model outputs post‐hazard (a) building‐level access to power, water, and communication, and (b) service facilities operational status, as shown in Figure 4. The operational status of facilities is used as input for the post‐hazard reassessment in the access model, as described in Section 3.1.4. While this study focuses on the impact of tropical cyclones, the model is adaptable to other hazards, given the necessary inputs, such as hazard loading and component‐specific fragility functions.

**FIGURE 4:**
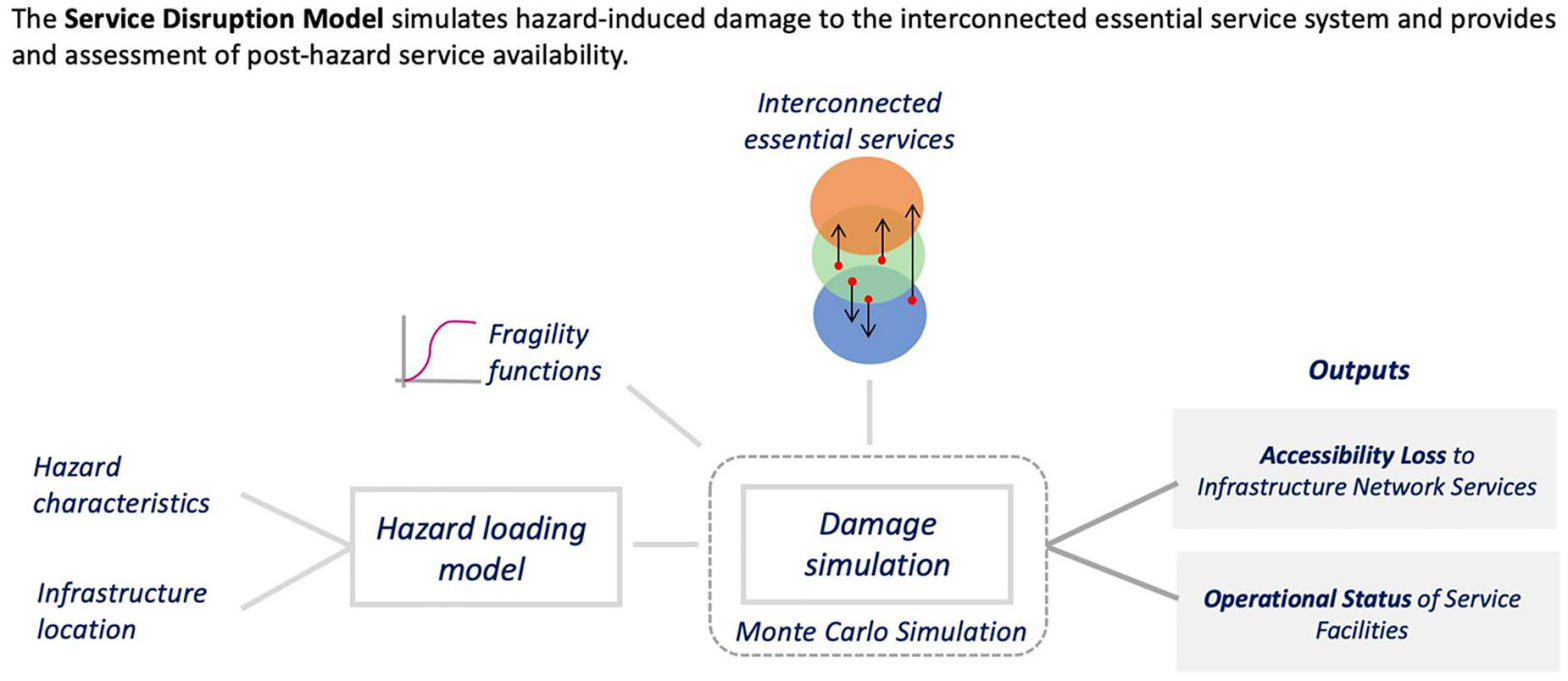
Service disruption model flowchart.

To estimate building‐level service loss, the model utilizes a parametric wind field model as the hazard loading mechanism to determine wind exposure at a component level. Component‐specific fragility functions are then applied to estimate the failure probability of each infrastructure component directly impacted by wind. However, fragility curves only describe component‐specific failures without capturing broader system‐level impacts. To address this, we integrated the component‐level damage estimates into a Monte Carlo simulation to capture the cascading effects across the interconnected service system.

The Monte Carlo simulation produces multiple replications of component damaged states, enabling probabilistic assessment of system‐wide disruptions. Each iteration (1) evaluates power network connectivity based on component failures, (2) simulates hydraulic dynamics to assess water supply disruptions caused by power‐dependent pump failures, and (3) evaluates communication network coverage loss due to both direct wind damage and power dependencies. This approach provides a probabilistic estimate of building‐level infrastructure network service loss, accounting for both direct damage and interdependencies across infrastructure systems.

In addition, each simulation iteration updates the operational status of service facilities based on network service availability. These updated statuses are then used to reassess building accessibility by recalculating the road network distance to the nearest operational facility, as described in Section 3.1.4.

##### 3.2.1.1 Hazard Loading Model

The hazard loading model used in this study is specific to tropical cyclone wind fields. However, the overall modeling framework can be adapted to other hazards by substituting the appropriate hazard‐specific loading models. Wind estimates are generated using a parametric wind field model based on Willoughby et al. (2006), implemented via the publicly available R package, *stormwindmodel*, which has been widely used in prior studies to estimate localized wind damage (Han, Guikema, and Quiring 2009; Han, Guikema, Quiring, Lee, et al. 2009; Tonn et al. 2016).

The *stormwindmodel* package produces wind fields based on the cyclone track data and maximum wind speed, typically recorded at 6‐h intervals. For each infrastructure component (e.g., buildings, poles, distribution lines, cellular towers), the model estimates surface‐level sustained winds, 3‐s gust winds, and the duration of winds exceeding a specified threshold (e.g., 20 m/s). These wind characteristics are calculated at the centroid of each component, or the midpoint for linear assets such as distribution lines.

To assess structural vulnerability, we utilized the maximum 3‐s gust wind speed at the surface level as input to component‐specific fragility functions. This enabled a probabilistic estimation of damage to individual infrastructure components.

##### 3.2.1.2 Fragility Functions

Empirical fragility curves are a widely used method for estimating the probability of a component reaching or exceeding a specific damage state under hazard loading (FEMA 2020). These curves are developed using fragility functions derived from expert judgment, experimental testing, or historical failure observations, and are both hazard‐ and component‐specific. As such, the likelihood of damage is a function of the hazard intensity and the component's inherent characteristics, including material properties and structural dimensions.

In this study, we utilized fragility functions to estimate the probability of wind‐induced failure for critical infrastructure components, including poles, distribution lines, and cellular towers, as a function of wind speed. Specifically, wind‐induced failure probabilities for distribution poles were estimated using the empirical fragility model developed by Bjarnadottir et al. (2013), with resistance parameters adopted from Li and Ellingwood (2006). Distribution line failures were modeled using the hurricane‐based empirical fragility functions developed by Quanta Technology (2009), while cellular tower failures were estimated using fragility functions derived from the nonlinear dynamic analyses of Bilionis and Vamvatsikos (2019). This approach represents the conventional methodology for modeling wind impacts on infrastructure. Detailed information on the fragility functions used in this analysis is provided in the Supporting Information.

##### 3.2.1.3 Damage Simulation

To address the uncertainties associated with hazard impacts on the interconnected essential services model, we integrated a Monte Carlo simulation. This probabilistic approach captures the stochastic nature of component failures and their cascading effects across the interconnected systems, accounting for both wind‐induced damage and system‐wide dependencies.

In each simulation iteration, poles and distribution line failures are generated based on wind loading and component‐specific fragility functions that directly estimate the probability of failure for each component. Once these primary failures are established, the power network's connectivity is evaluated, and all downstream components that rely on the failed poles and distribution lines are identified and classified as non‐operational.

The simulation then propagates power network disruptions into the water and communication networks. In the water network, pump failures are determined by their dependency on failed power poles. To assess the resulting water service disruptions, a pressure‐driven hydraulic simulation is conducted to assess water flow dynamics and identify buildings that lose service due to insufficient pressure.

For the communication network, failures of cellular towers and antennas are modeled based on both direct wind impacts and power dependencies. In each Monte Carlo realization, components are classified as failed when a uniformly distributed random sample falls below the failure probability estimated by the fragility function. Coverage areas are dynamically updated to reflect damaged components, and buildings outside these areas are identified as having lost communication access.

The Monte Carlo simulation was executed for 400 iterations, a value selected to ensure statistical convergence using a 95% confidence interval with a total width of 0.10. The required number of iterations was determined using the following equation (Law 2014):

$$N\geq {\left(\frac{2cS}{w}\right)}^{2}$$

where c is the *Z*‐score for the desired confidence level (1.96 for 95%), S is the standard deviation of the estimated metric, and w is the desired confidence interval width (0.10). This ensures that the estimated probabilities of buildings losing access to one or more essential services (power, water, or communication) remain within specified precision bounds.

In each iteration, the model recorded failed components across the power, water, and communication networks and identified buildings without access to one or more services. These outputs were aggregated to estimate the probability of infrastructure service loss at the building level.

For the WDS, the only source of uncertainty arises from the failure of power poles, as the pumps with backup power systems were known from available data (AAA 2015). Therefore, one hydraulic simulation was performed per power network realization (N=400).

In contrast, the communication model incorporated additional uncertainty due to limited information on backup power availability for towers and antennas. To address this, backup systems were randomly assigned in each simulation. For each power network realization, 300 independent communication network simulations were run. The operational status of each antenna or tower was then determined using a majority‐vote rule: if the antenna failed in more than 50% of the simulations, it was classified as non‐operational; otherwise, it was considered functional. This approach accounts for uncertainty in power backup availability and its cascading impact on communication service reliability.

##### 3.2.1.4 Accessibility Loss Metrics

The overall model produces two primary metrics to quantify accessibility loss. The first measured building‐level loss of access to infrastructure services by estimating the probability that a building loses power, water, and communication. This is calculated by aggregating binary access indicators from the Monte Carlo simulation. For each iteration, the model assigns $a_{i,j}=1$ if building i maintains access to the service j, and $a_{i,j}=0$ if access is lost, where j=1 for power, j=2 for water, and j=3 for communication. The probability that building i loses access to service j is computed as the complement of the expected value of $a_{i,j}$ across all simulation iterations:

$$P\;\left(\;a_{i,j}=0\right)=1-\frac{1}{N}\sum_{n=1}^{N}a_{i,j}^{\left(n\right)}$$

where N is the total number of simulation iterations, and $a_{i,j}^{\left(n\right)}$ denotes the binary access status of building i to service j in iteration n.

The second metric, Estimated Increase in Travel Distance (EITD), quantifies the difference between post‐hazard and pre‐hazard travel distances due to service facility disruptions. EITD is calculated using the operational status of service facilities, which is determined in each Monte Carlo iteration based on infrastructure network availability. For each iteration, facility availability is updated and building‐level travel distances to the nearest operational facility are recalculated, following the methodology in Section 3.1.4.

The EITD for building i and service j is computed as:

$$\;\mathrm{EIT}D_{i,j}=\frac{1}{N}\;\sum_{n=1}^{N}D_{i,j}^{\left(n\right)}-d_{i,j}$$

where $D_{i,j}^{\left(n\right)}$ is the post‐hazard distance from building i to the nearest operational facility of type j in iteration n, $d_{i,j}$ is the pre‐hazard baseline distance, and N is the total number of simulation iterations.

If no operational service facility of type j is available within the study area during a given iteration, $D_{i,j}^{\left(n\right)}$ is set as the distance to the nearest facility in a neighboring community. This assumption represents the minimum additional travel distance residents would need to cover, under the premise that the service in neighboring communities remains operational and accessible.

The methodology described above provides a scalable and data‐driven approach to evaluate loss of access by integrating infrastructure network performance, service facility availability, and building‐level impacts. The following section presents the application of the AccES framework through a case study in Cayey, Puerto Rico, demonstrating its effectiveness in assessing post‐disaster accessibility following a simulated tropical cyclone.

## 4 Case Study

This section applies the proposed AccES framework to Cayey, Puerto Rico, a municipality located in the island's central mountainous region (Figure 5). Cayey was selected due to its historical exposure to tropical cyclones and its geographical susceptibility to meteorological and hydrological hazards. Past storms have caused widespread infrastructure damage, extensive vegetation loss, and prolonged power outages.

**FIGURE 5:**
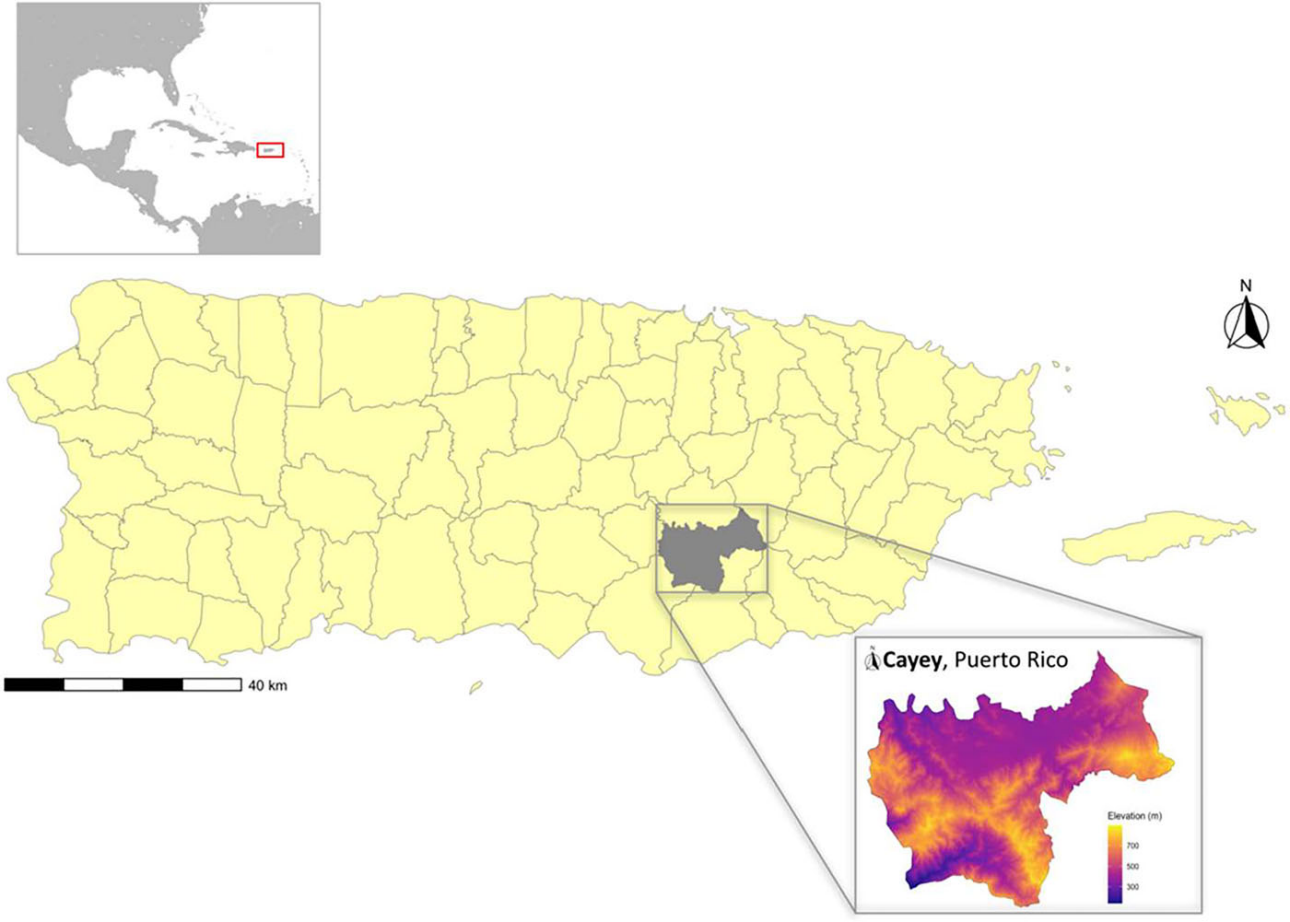
Geographical location of Cayey, Puerto Rico.

As of 2023, Cayey had a population of 41,231 (U.S. Census Bureau 2023). The municipality faces significant socioeconomic challenges, with a median household income of $28,461 and a poverty rate of 37.9%. It is also highly car‐dependent, with 93% of the population relying on personal vehicles for daily transportation. In addition, 23% of the residents are aged 65 or older, and 32% have at least one disability, making them more susceptible to mobility limitations and health risks during service disruptions.

Cayey's terrain ranges in elevation from 284 to 840 m above sea level(U.S. Geological Survey [USGS] 2019). These elevation differences are particularly important for water distribution, as pumping systems are required to deliver water to higher‐elevation areas. In the event of power outages, pump failures can lead to significant water disruptions, disproportionately affecting residents who rely on mechanical pumping rather than gravity‐fed distribution.

### 4.1 Essential Services in Cayey, Puerto Rico

The identified essential services for this community, based on interviews with community stakeholders, were power, water, communication, transportation, hospitals, grocery stores, gas stations, childcare centers, schools, elder care facilities, and veterinary services. It is worth noting that the stakeholder‐driven list was broader than typical predefined essential service frameworks, incorporating locally relevant services such as childcare that are often not explicitly included. Geographic data for these services are publicly available for most US regions through open‐source platforms such as OSM and multiple local government data repositories (Haklay and Weber 2008). For instance, the geographical locations of infrastructure and facilities were obtained from OSM using relevant tags such as “power = substations” for substations, “amenity = fuel” for gas stations, and “shop = supermarket” for grocery stores. These data were further validated using satellite imagery from Google Maps, government datasets, and on‐site validation. Direct links to data sources and the OSM code are provided in the Supporting Information. Figure 6 shows the spatial distribution of service facilities, most of which are concentrated in the urban center of the municipality.

**FIGURE 6:**
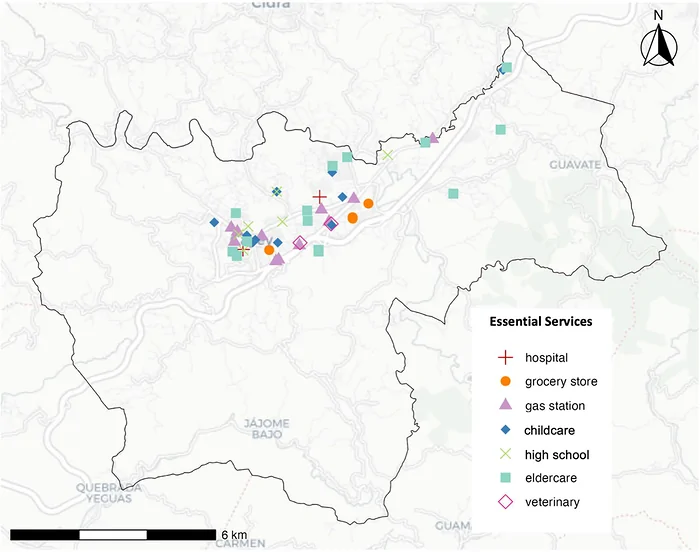
Spatial distribution of service facilities in Cayey, Puerto Rico.

To represent childcare services, the analysis focused on federally funded Head Start centers, which offer early education, childcare, and health support for children from low‐income households. While informal providers exist, only officially registered centers were included. Similarly, eldercare services were limited to licensed nursing homes, which serve a vulnerable population requiring continuous medical and personal care. In the case of grocery stores, only major grocery retailers were included, as they serve as the primary food sources within the community. Other establishments, such as convenience stores and restaurants, were excluded due to their limited inventory and reduced capacity to maintain food supply during disruptions.

Details on the locations of infrastructure network components are provided in the Supporting Information, including substations for the power network, WTPs, storage tanks, pumps, and valves for the water network, and communication towers and antennas.

### 4.2 Accessibility Baseline

The travel distance from each building to the nearest operational service facility was calculated for pre‐hazard conditions, assuming services are fully operational before the hazard event. Figure 7 shows the accessibility maps and the cumulative distribution plots of travel distances for four essential service facilities: hospitals, childcare centers, grocery stores, and gas stations. Additional results for veterinary clinics, eldercare facilities, and schools are provided in Supporting Information.

**FIGURE 7:**
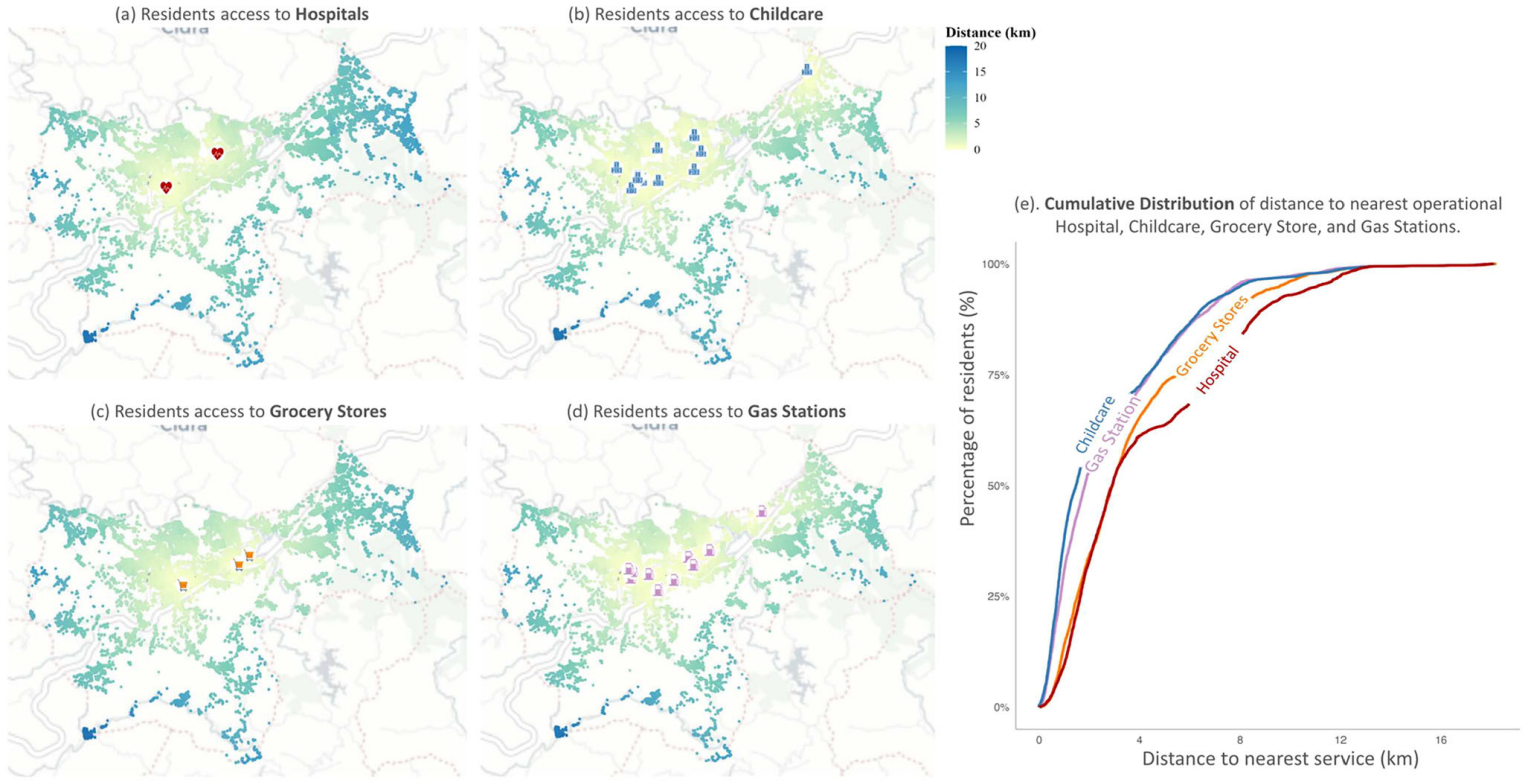
Accessibility maps and the cumulative distribution plots of travel distances for four essential service facilities pre‐hazard: hospitals, childcare centers, grocery stores, and gas stations in Cayey, Puerto Rico.

The maps use a light‐to‐dark blue gradient, with darker shades indicating greater travel distances to the nearest facility. Distances for our case study community range from 0 to 20 km, highlighting spatial disparities in access to services pre‐hazard. As shown in the cumulative distribution plot (Figure 7e), approximately 40% of the population resides more than 4 km away from each service type. These differences in baseline accessibility reflect the underlying spatial structure of the community, with rural areas consistently exhibiting longer travel distances compared to urban areas due to lower facility density and greater geographic dispersion.

Among the services analyzed, hospitals exhibit the highest travel distances, with around 25% of the population residing over 8 km away from the nearest hospital, which can have serious implications for emergency response times and healthcare access. While accessibility analyses were conducted for all essential service facilities, the remainder of this chapter will focus on hospital access due to its critical role in life‐saving interventions. Additional analysis is included in the Supporting Information.

### 4.3 Hazard Loading Model

For this assessment, the impact of a Category 2 tropical cyclone, based on the Saffir–Simpson Hurricane Wind Scale, was simulated on Cayey, Puerto Rico. A parametric wind field model was used to estimate hazard loading, in terms of wind speed, at the component level for both buildings and critical infrastructure. Wind speed values ranged between 40 and 43 m/s, as shown in Figure 8. These wind estimates were integrated with the fragility functions described in the Supporting Information to assess the potential damage state of critical infrastructure components.

**FIGURE 8:**
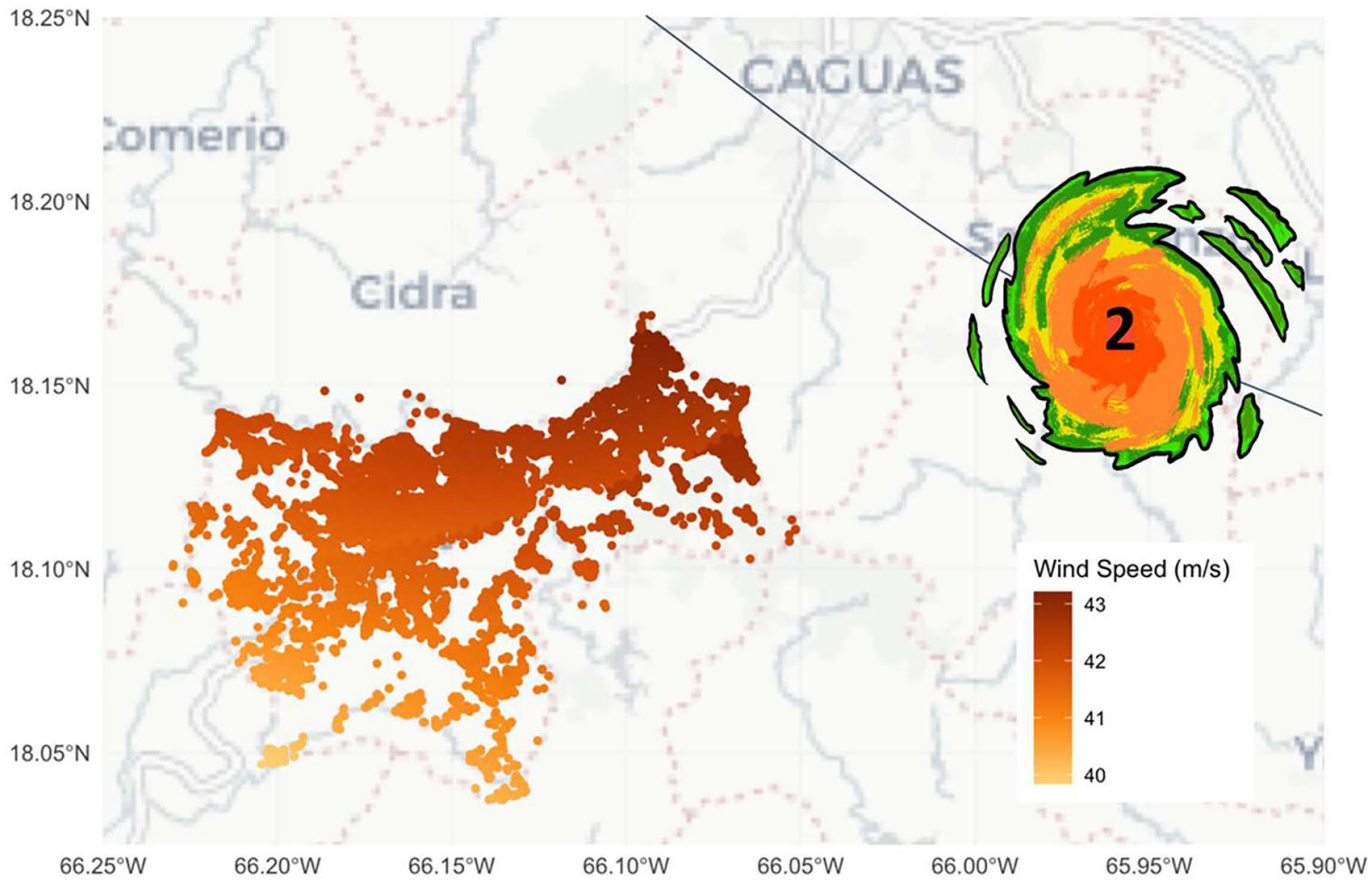
Wind loading: 3‐s gust wind estimates (m/s) at the component level.

To provide context for a more extreme event, a comparative simulation was conducted for a Category 4 tropical cyclone. In this scenario, all essential services within the municipality failed, consistent with disruptions observed following Hurricane Maria in 2017 (Chandra et al. 2021; National Institute of Standards and Technology [NIST] 2021).

This analysis focuses on the Category 2 tropical cyclone scenario, as it allows for the examination of partial service disruptions. This scenario demonstrates how accessibility is affected when some facilities remain operational while others close. Although this study focuses on tropical cyclone impacts, the methodology is adaptable to different hazard types and intensities, given the hazard loading and fragility functions.

### 4.4 Accessibility Loss Estimates

#### 4.4.1 Accessibility Loss to Infrastructure Networks

Figure 9 illustrates the probability of losing access to essential infrastructure services, where blue indicates low risk and red represents high risk of accessibility loss. The spatial distribution of these probabilities provides insights into how different regions of the municipality are affected by disruptions in the power, water, and communication networks.

**FIGURE 9:**
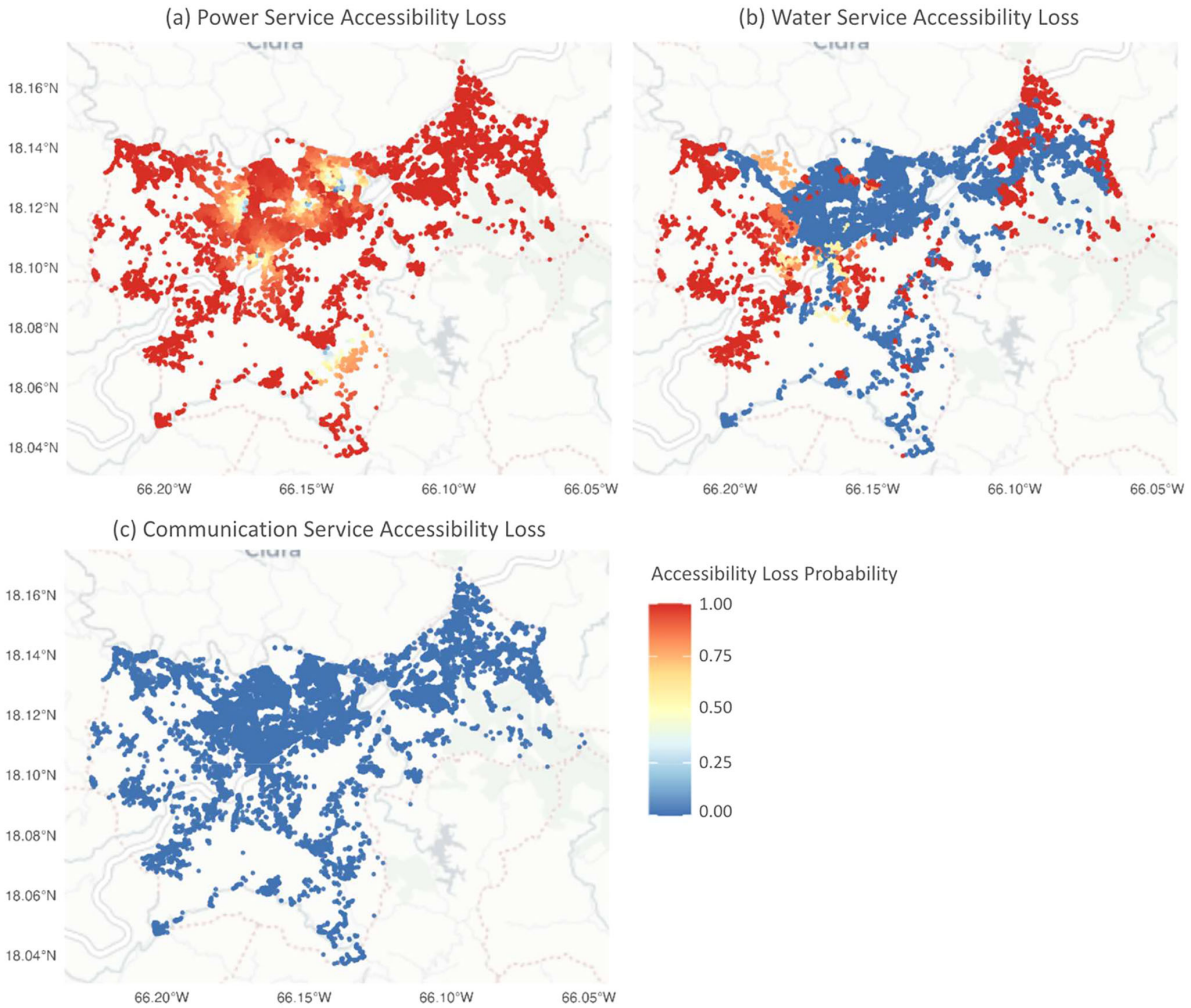
Infrastructure networks’ accessibility loss probability at a building level.

FIGURE 9 (a) represents the power network accessibility loss. Most areas in the municipality face a high probability of losing power, primarily due to the radial structure of the distribution system. As the distance from the substation increases, so does the likelihood of service disruption. Areas closer to substations exhibit lower outage risk, while more remote, rural regions have significantly higher probabilities of power loss.

Figure 9b represents the water network accessibility loss, which is significantly influenced by elevation and proximity to WTPs. The urban center, located at lower elevations, shows a lower probability of losing access to water since it primarily relies on gravity‐fed water distribution, and urban pump stations, as indicated by the utility company, tend to have backup power systems. In contrast, higher elevation rural areas, especially those farther away from WTPs, show a higher probability of losing access to water, mainly due to their reliance on pump stations. In the model, gravity‐fed distribution is assumed to operate independently of electricity availability, whereas pump‐dependent supply is sensitive to power disruptions. This highlights the disproportionate impact on rural areas of the municipality, which are more dependent on the power infrastructure. The pump stations with backup power generators are included in the Supporting Information.

The communication network remains largely unaffected under the simulated Category 2 tropical cyclone scenario, as shown in Figure 9c, with a low probability of accessibility loss across the municipality. This is attributed to the high redundancy in the network and the availability of backup power systems, such as batteries and generators, for cell towers and antennas. The results are consistent with historical observations, which indicate that low‐intensity tropical cyclones typically do not experience significant damage or service interruptions in the communication network due to their built‐in resilience (Khoury 2025). In contrast, high‐intensity cyclones (e.g., Category 4 or 5 hurricanes) are known to cause significant structural damage, leading to more severe service disruptions (GAO 2021b).

#### 4.4.2 Accessibility Loss to Service Facilities

This section presents the EITD results for hospitals as a demonstration of essential service accessibility under post‐hazard conditions. Although this analysis focuses on hospitals, the same methodology is applicable to other critical service facilities, with results for additional services provided in the Supporting Information.

Cayey has two hospital facilities, a primary hospital, and a secondary urgent care center, both located within the urban area. For this analysis, we evaluated two scenarios: (a) neither hospital has access to backup power, and (b) only the primary hospital is equipped with backup power, while the secondary facility remains disrupted during outages. These scenarios allow us to evaluate how backup systems impact building‐level accessibility.

In both scenarios, if no hospital in Cayey is operational during a simulation iteration, the model assumes residents travel to the nearest operational hospital outside the municipality. For this analysis, the alternative facility is located in the neighboring municipality of Caguas, approximately 25 km from Cayey's urban center. This alternative facility was selected based on its capacity, regional importance, and proximity through main roadways, which are prioritized in post‐disaster road clearance operations. While only a single alternative facility is explicitly modeled in this case study, the framework is designed to accommodate multiple external facilities if available.

Figure 10 represents the spatial distribution of EITD across buildings for both scenarios. Darker shades indicate a greater increase in travel distance to an operational hospital, representing higher burdens on the affected population. Hospital facilities (i.e., the two modeled hospitals and the alternative facility) that were non‐operational in more than 50% of simulation runs are shown in red, while those assumed to remain operational due to backup systems are shown in green.

**FIGURE 10:**
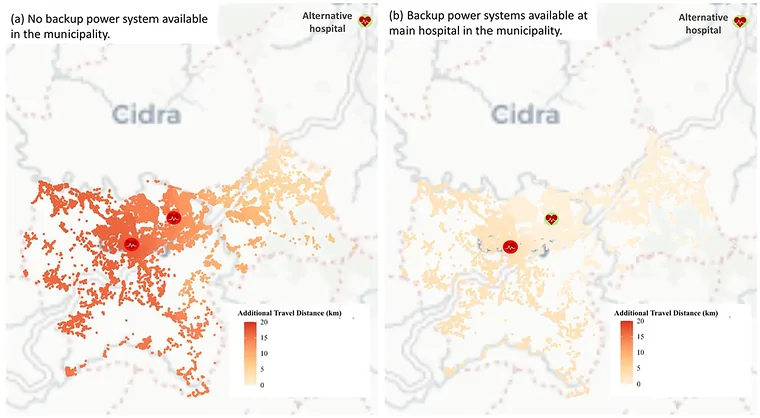
Spatial distribution of EITD to hospitals at the building level under two post‐disaster scenarios: (a) neither hospital has access to backup power generation, and (b) only the primary hospital is equipped with a backup power system. In this analysis, two hospitals are located within the municipality, and an alternative facility is located in the neighboring municipality of Caguas, Puerto Rico, shown in the top‐right corner of the map (labeled “Alternative hospital”).

Scenario (a) results, shown in Figure 10a, show that the absence of backup systems at both hospitals results in widespread service unavailability, with over 75% of the simulations indicating that neither hospital is operational, forcing residents to travel outside of the municipality. As a result, in most iterations, the additional travel distances range from 1 km in the northeast to over 20 km in the western and southern sectors, disproportionately impacting rural areas with pre‐existing limited access to healthcare services.

Scenario (b) results, shown in Figure 10b, highlight the benefits of equipping the primary hospital with backup power. In most simulation iterations, the hospital remains operational, significantly reducing travel burdens, especially in the central and western parts of the municipality. The availability of in‐municipality care can drastically reduce delays in emergency response, particularly for vulnerable populations such as the elderly or those without access to personal vehicles.

Figure 11 shows the cumulative distribution of travel distances to the nearest hospital under each scenario, compared to pre‐hazard conditions. Under pre‐hazard conditions (blue curve), approximately 95% of residents are located within 10 km of a hospital. In Scenario (a) (red curve), over 50% of the population must travel more than 15 km, with some traveling distances exceeding 30 km. Implementing a backup power system to the main hospital in the municipality (green curve) significantly reduces travel burdens, bringing the distribution closer to pre‐hazard conditions, with around 90% of residents within 10 km of an operational hospital.

**FIGURE 11:**
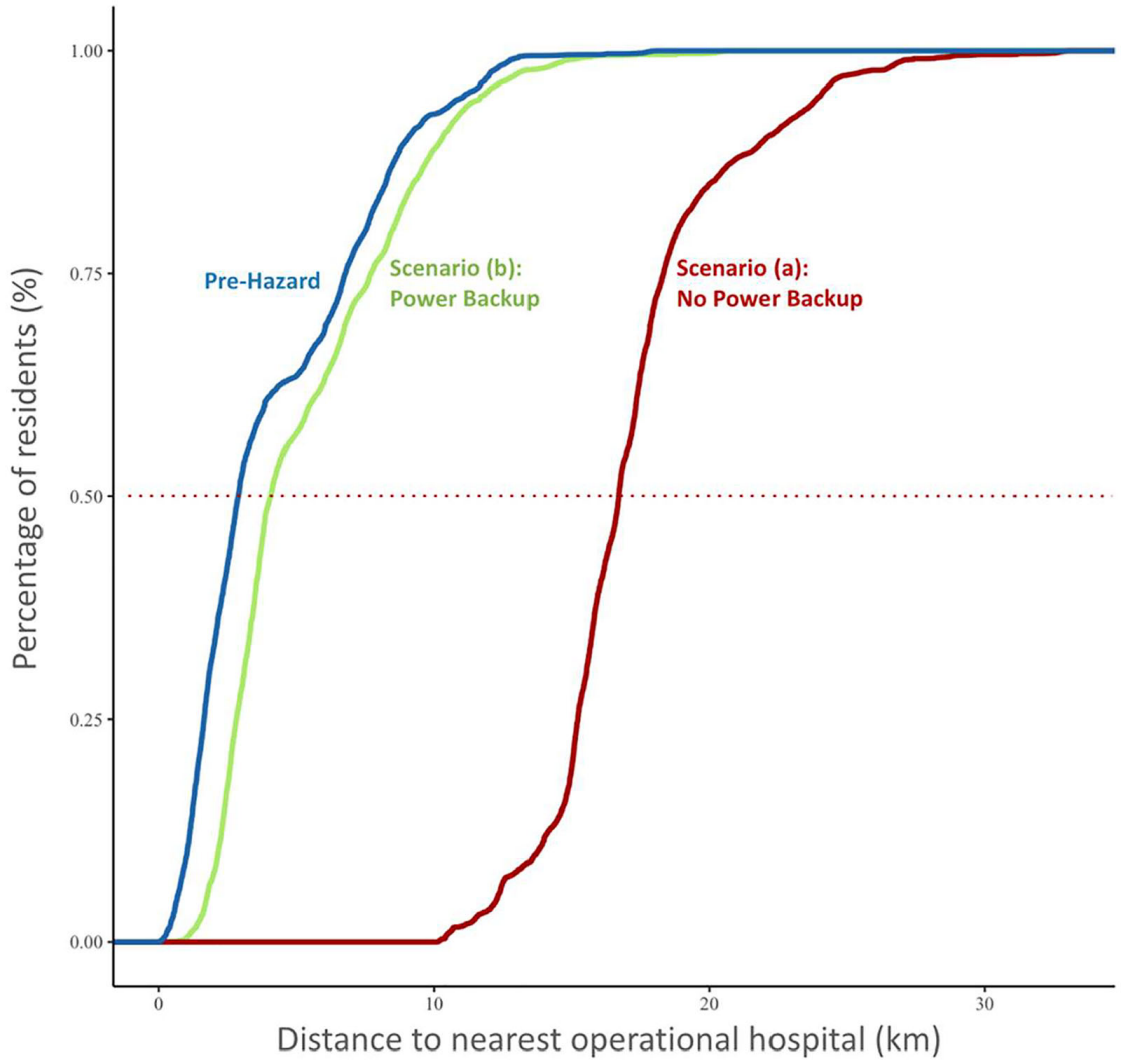
Infrastructure networks’ accessibility loss probability at a building level.

#### 4.4.3 Accessibility Loss to Service Facilities by Census Tract

The results of this section demonstrate the potential of integrating the accessibility analyses with community‐level vulnerability data to support targeted and equitable disaster response planning. For this assessment, we incorporate the U.S. Census Bureau Community Resilience Estimates (CRE) for Puerto Rico (U.S. Census 2024), which provides tract‐level estimates of the population's adaptive capacity in the event of a disaster. The CRE index indicates the percentage of individuals within each census tract that have three or more vulnerability factors, including poverty status, number of caregivers in the household, unit‐level crowding, education level, employment, disability status, healthcare coverage, age (65+), vehicle access, and broadband internet access.

Several CRE indicators align with the vulnerable populations identified through interviews with community leaders in this study, notably poverty, older age, and disability status. The CRE index is consistent with prior research on distributional‐demographic equity, which highlights the importance of demographic characteristics, such as age, income, disability, education, employment, and household composition, when assessing disparities in infrastructure resilience and service accessibility (Coleman et al. 2024).

However, it is worth noting the limitations of the CRE index. The metric treats all vulnerability factors as equally weighted and assumes that any three factors result in “high vulnerability,” regardless of which three. In practice, some factors, such as lack of transportation, may have a much greater impact on disaster resilience than others, like lack of broadband access or vice versa. The lived experiences and adaptive capacities associated with each vulnerability factor can vary greatly depending on local conditions and community context. Therefore, while the CRE index offers a valuable starting point for identifying vulnerable areas in the community, it should be used cautiously and, when possible, supplemented with community‐specific insights and additional data. For purposes of this demonstration, we incorporated the estimations as defined by the U.S. Census Bureau.

Figure 12 presents box plots showing the distribution of travel distances to the nearest operational hospital by census tract, comparing baseline (pre‐hazard) conditions with Scenario (a), in which neither hospital is equipped with a backup power. Each tract is represented by two box plots: the lighter shade box plot on the left represents pre‐hazard access, and the darker box plot on the right represents post‐hazard access. Box plot colors correspond to the percentage of residents with at least three social vulnerability (SoVI) factors, as defined by the CRE index, with darker tones indicating higher vulnerability levels, as defined by the U.S. Census Bureau.

**FIGURE 12:**
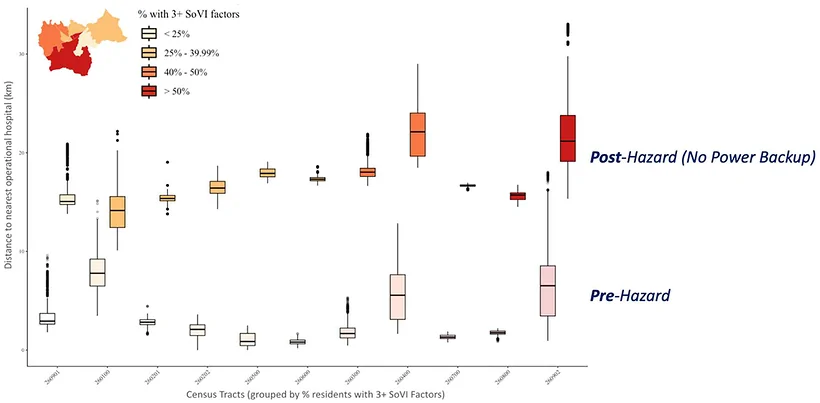
Boxplots of the distribution of travel distances to the nearest operational hospital by census tract for pre‐hazard baseline conditions and Scenario (a), in which neither hospital is equipped with backup power.

Figure 12 highlights the compounding impact of service disruptions on socially vulnerable areas. For example, Census Tract 260902, located in the rural, mountainous southwest of Cayey, shows limited hospital access both before and after a hazard. This tract also exhibits a high concentration of socially vulnerable residents, as defined by the CRE index, where over 28% of the population is aged 65 or older, 67% of minors live below the poverty line, and 26% of adults do not have a high school diploma. Furthermore, this area has historically experienced slower restoration times, especially of the road network, following major natural hazard events. While road impact and recovery are not modeled in this analysis, such disruptions and slow recovery may further exacerbate this census tract's isolation and difficulty accessing critical services during post‐disaster recovery.

These overlapping burdens suggest that residents in Tract 260902 face elevated risks of delayed medical care following a disaster, due to both geographic barriers and limited adaptive capacity. Insights like these are critical for equity‐focused emergency planning. Emergency planners could leverage this integrated analysis to prioritize areas such as Tract 260902 for temporary medical units or mobile health services in the aftermath of a disaster.

The results also indicate heterogeneity across census tracts in the magnitude of change between pre‐ and post‐hazard conditions. For example, Tract 260100 exhibits a smaller increase in travel distance relative to its baseline conditions compared to Tract 260902, suggesting lower sensitivity to service disruptions. This indicates that vulnerability is not only driven by baseline access, but also by the relative deterioration in access following hazard events.

## 5 Conclusion

This study introduces the **AccES** framework, a novel, data‐driven, people‐centered, equity‐based approach designed for evaluating building‐level loss of access to essential services following a hazard event. To our knowledge, AccES is the first framework to rely entirely on publicly available datasets, supplemented with local stakeholder input, to generate spatially explicit representations of interconnected essential service systems and assess access to them before and after a hazard event. The framework accounts for disruptions to both infrastructure networks and service facilities, enabling quantification of loss of access across essential services.

AccES recognizes that equitable access to essential services will lead to more resilient communities, as outlined by Coleman et al. (2020). Equity‐oriented approaches are necessary because if resilience strategies fail to account for the disproportionate impact, particularly on marginalized populations, they risk reinforcing or exacerbating existing vulnerabilities. This framework incorporates multiple dimensions of equity, as identified in Coleman et al. (2020).

First, it supports distributional‐demographic equity, which refers to the fair distribution of resources and services considering the differing needs and vulnerabilities of specific demographic groups. By providing building‐level assessments of accessibility to essential services, the framework enables the integration of micro‐level demographic data, when available, to evaluate how specific groups experience access loss. Second, the framework integrates distributional‐spatial equity, which focuses on the equitable distribution of services across geographic areas. The AccES model produces spatially explicit accessibility maps that highlight disparities in access across geographic areas and allow us to identify service desert areas before and after a hazard.

Procedural equity, which involves inclusive engagement with stakeholders and community members, is partially addressed. While the model integrates local knowledge through interviews and surveys to identify essential services, service dependencies, and backup strategies, ensuring the model reflects localized needs and priorities, stakeholders were not directly involved in the research planning process. Finally, AccES contributes to capacity equity by aligning service access with the needs of households. It provides a quantifiable access metric, and the model can be further used to evaluate multiple adaptation strategies, allowing decision‐makers to consider both the availability of services and the varying ability of households to utilize them.

To demonstrate some of the framework's potential applications, the AccES framework was implemented in a case study in Cayey, Puerto Rico, simulating a Category 2 tropical cyclone. Although this analysis centers on hurricane‐related disruptions, the framework is hazard‐agnostic and can be adapted to other hazards (e.g., earthquakes, wildfires), provided the hazard loading and components' fragility functions are available. As it relies on publicly available data, AccES can be applied to any US community.

Several limitations are worth acknowledging. The model quantifies access as physical proximity through road network distances. While this is a widely used metric, it captures only one dimension of access and does not incorporate other critical factors such as acceptability, affordability, adequacy, or awareness (Penchansky and Thomas 1981; Saurman 2015). This simplification reflects current data constraints, as many relevant indicators are only available at aggregated spatial scales that are incompatible with the building‐level resolution of this analysis. In addition, an alternative and potentially more realistic metric would leverage travel time, accounting for road types, speed limits, and mode‐dependent travel speeds. Furthermore, the model assumes that the transportation network is fully operational in post‐disaster conditions. In reality, disasters often damage or obstruct the transportation infrastructure, impacting accessibility to services and potentially isolating sectors in the community. While such an impact is not currently modeled, future work could integrate road disruptions to enhance the results.

In addition, limited data on the availability of backup power, particularly in the communications sector, required the use of simplifying assumptions. As better data become available, these can be refined. Moreover, the current model represents only the immediate post‐disaster period. Future extensions could incorporate recovery dynamics to assess service restoration over time. Finally, while the CRE index provides a useful proxy for social vulnerability, the use of it as a single composite measure reduces the complexity of social conditions and may not fully reflect the diversity of lived experiences or place‐specific sources of vulnerability.

The AccES framework has wide‐ranging practical applications. For instance, emergency managers can use the results to identify high‐risk areas and strategically locate mobile clinics, water distribution points, or temporary generators. Results from this model can also inform infrastructure investments, such as prioritizing backup systems at essential service facilities or reinforcing vulnerable infrastructure network components. In addition, by integrating accessibility loss with adaptive capability indicators (e.g., from the U.S. Census CRE), the model enabled the development of more equitable policies aimed at reducing disproportionate burdens on marginalized populations. Communities can also leverage AccES results in applications for federal mitigation or resilience funding by clearly demonstrating spatial and demographic disparities in service access. Ultimately, AccES enables more data‐informed, targeted, and equitable disaster preparedness and recovery strategies.

## Author Contributions

**Zaira Pagan‐Cajigas**: Developed the AccES framework, led the design, coding, and implementation of the models; processed and analyzed the data; interpreted the results; created visualizations and tables; and led the writing and revision of the manuscript. **Seth Guikema**: Supervised the research and framework development, provided conceptual and technical guidance, and contributed to the writing and revision of the manuscript.

## Supporting information




**Supporting information**: risa70373‐supp‐0001‐SuppMat.docx
