# Supplementary material for: AccES: A Framework for Predicting Building‐Level Loss of Accessibility to Essential Services Post‐Disasters Based on Publicly Available Information
Source: Risk Anal. 2026 Oct 7;46(11):e70373. doi: 10.1111/risa.70373 (PMC13643670; doi:10.1111/risa.70373)
Supplement: Supplementary file 1 [file RISA-46-0-s001.docx]

Supplemental Material for

**AccES: A Framework for Predicting Building-Level Loss of Accessibility to Essential Services Post-Disasters Based on Publicly Available Information.**

1. **Datasets**

**Table S1. Dataset and Links for Cayey, Puerto Rico**

| **Data** | **Link(s)** |
| --- | --- |
| Road Network | <https://catalog.data.gov/dataset/tiger-line-shapefile-current-county-cayey-municipio-pr-all-roads> |
| Building Footprints | <https://data.humdata.org/dataset/hotosm_pri_buildings>; <https://catalog.data.gov/dataset/tiger-line-shapefile-2022-county-cayey-municipio-pr-address-range-feature> |
| Elevation | <https://apps.nationalmap.gov/downloader/> |
| Substations | <https://overpass-turbo.eu/>; <https://gis.pr.gov/descargaGeodatos/Infraestructuras/Pages/Electricidad.aspx> |
| Cellular Towers and Antennas | <https://opencellid.org/#zoom=15&lat=18.1029&lon=-66.164> |
| Water System Components | <https://gis.pr.gov/descargaGeodatos/Infraestructuras/Pages/Agua.aspx> |
| Childcare | <https://headstart.gov/center-locator?latitude=18.221&longitude=-66.590&address&city&zip&state=PR&redirect=eclkc> |
| Hospitals | <https://www.ahd.com/states/hospital_PR.html> ; <https://data.humdata.org/dataset/hotosm_pri_health_facilities> |
| Schools | <https://nces.ed.gov/ccd/schoolsearch/school_list.asp?Search=1&County=Cayey%20Municipio&State=72>; <https://buscatuescuela.dde.pr/> |
| Fragility Functions | <https://zenodo.org/records/10203846> |
| Community Resilience Estimates | <https://www.census.gov/programs-surveys/community-resilience-estimates/data/cre-pr.html> |

2. **Overpass Turbo Codes**

**Listing S1**: Overpass Turbo query for substations in Cayey.

**Listing S2**: Overpass Turbo query for gas stations in Cayey.

**Listing S3**: Overpass Turbo query for grocery stores in Cayey.

3. **Fragility Functions**

***3.1 Distribution Poles***

Poles are critical components of power distribution networks and are particularly vulnerable to wind loading (Nirandjan et al. 2024). Multiple studies have developed fragility functions to model wind-induced failures of poles based on factors such as material type (e.g., wood, steel), design class, height, and age (Quanta, 2009; Bjarnadottir et al., 2013; Shafieezadeh et al., 2014; Teoh et al., 2019). For this study, we adopted the empirical fragility function presented by Bjarnadottir et al. (2013) to estimate the probability of pole failure under wind loading. This function assumes new pine poles with a design class of 4 and a height of 13.7 m. It is important to note that this equation only accounts for wind impact and does not consider potential failures caused by trees or debris impacts. The failure probability of poles ($P_{failure, pole})$ due to wind loading is calculated using the following equation:

$$P_{failure, pole}= \Phi\left( \frac{ln\left( \frac{V}{m_{R,pole}} \right)}{\sigma_{R,pole}} \right)$$

where $V$ is 3-second gust wind speed (m/s); $m_{R,pole}$ is the median resistance of the pole, defined as 51.1547, as per Li and Ellingwood (2006); $\sigma_{R,pole}$is the logarithmic standard deviation of the resistance, set as 0.146 as per Li and Ellingwood (2006); and Φ represents the cumulative distribution function of the standard normal distribution.

Figure S1. Fragility curve for new pine poles with a design class of 4 and a height of 13.7 m.

***3.2 Distribution Lines***

Distribution lines are predominantly impacted by the direct impact of falling trees and flying debris, as demonstrated by Cerrai et al. (2019). These factors are highly correlated with wind loading, allowing us to model the impact on distribution lines as a function of wind speed, similar to the approach used for poles. This study adopts the fragility function developed by Quanta Technology (2009), based on data collected across six hurricane events. This approach accounts for multiple factors that may lead to distribution line failure, including tree coverage and wire size. The probability of failure for distribution lines ($P_{failure, lines})$is calculated using the following equation:

$$P_{failure, line}= \alpha V^{b}$$

where $V$ is the 3-second gust wind speed (m/s), and $\alpha$ and $b$ are empirically derived tuning parameters. Using the historical data from Florida power infrastructure, Quanta Technology (2009), defined $\alpha$ equal to $8({10}^{-12})$ and $b$ equal to 5.1731.

While this fragility function is based on Florida's power infrastructure, its application to Puerto Rico is appropriate for several reasons. First, the main causes of distribution line failure, which are wind-induced tree fall and debris impact, are similar across hurricane-prone subtropical and tropical regions. Second, Puerto Rico exhibits comparable vegetation density, urban-forest interface, and exposure to extreme wind events. Therefore, despite potential differences in terrain or vegetation management practices, the function provides a reasonable baseline for estimating wind-related failures in Puerto Rico’s distribution network. Nevertheless, localized calibration would enhance the precision of the model and is recommended for future work.

Figure S2. Fragility curve for distribution lines.

***3.3 Cellular Towers***

Cellular towers have been impacted severely by high-intensity wind scenarios (Tian et al., 2020). Despite the critical nature of this infrastructure, there are limited well-developed fragility functions that model cellular tower damage states due to wind speed. A few existing studies have developed fragility functions, mainly based on structural properties, material strengths, and tower geometry.

This analysis utilized fragility functions developed by Bilionis and Vamvatsikos (2019), who conducted a nonlinear dynamic analysis to assess the wind-induced failure probabilities of steel lattice cellular towers equipped with four antennas. Their study evaluated both ice and non-ice conditions; however, for our analysis, we focused on the non-ice conditions functions, assuming a 48-meter-tall steel lattice tower, a design commonly used in telecommunications infrastructure.

The probability of failure of cellular towers ($P_{failure, towers})$is calculated using the following equation:

$$P_{failure, tower}= \Phi\left( \frac{ln\left( \frac{V}{m_{R,tower}} \right)}{\sigma_{R,tower}} \right)$$

where $V$ is 3-second gust wind speed (m/s); $\Phi$ represents the cumulative distribution function of the standard normal distribution; $m_{R,tower}$ is the median resistance of the cellular tower, defined as 37.62; $\sigma_{R,tower}$is the logarithmic standard deviation of the resistance, set as 0.144. The values for $m_{R,tower}$ and $\sigma_{R,tower}$were obtained from experimental studies and literature on steel lattice towers, as referenced in Bilionis and Vamvatsikos (2019).

Figure S3. Fragility curve for a 48-meter steel lattice communication tower.

4. **Individual Infrastructure Networks**

***4.1 Power Network***

The locations of substations are publicly available for most U.S. cities through open-source platforms such as OpenStreetMap (Haklay and Weber, 2008). For the substations, we utilized tags such as “*power = substations*” and cross-verified the location of these components using satellite imagery from Google Maps, governmental datasets (Table S1), and on-site validation. The spatial distribution of substations in Cayey (Figure S4), illustrated by blue dots, highlights that the majority are located within the municipality’s urban area.

Figure S4. Substation location in Cayey, PR.

***4.2 Water Network***

The locations of WTP(s) and storage tanks are publicly available for most U.S. cities through open-source platforms such as OpenStreetMap (Haklay and Weber, 2008). For the case study in Cayey, Puerto Rico, initial data on water infrastructure components were obtained directly from the municipal government (AAA, 2015), including the locations of WTPs, storage tanks, pumps, valves, and segments of the pipeline network, shown in Figure S5. These data were supplemented and cross-validated using OpenStreetMap. WTPs were identified using the tag “*water_works*” and storage tanks were identified using the tags “*water_tower*” and “*storage_tank*” with the attribute “*content=water*”. To improve accuracy, all OpenStreetMap identified infrastructure components were cross-referenced with high-resolution satellite imagery and mapping platforms such as Google Maps. While the OpenStreetMap data for Cayey proved to be largely accurate, the quality and completeness of OpenStreetMap vary by location, and similar efforts in other municipalities may require more extensive validation.

Figure S5. The water network component’s location in Cayey, PR.

Information on pump stations equipped with backup generators was obtained directly from the local water distribution utility. These pumps, which are essential for ensuring the continuity of water service during power outages, are shown in Figure S6.

Figure S6. Pump stations with backup power generators in Cayey, PR.

***4.3 Communication Network***

The locations of cellular towers and antennas are publicly available through open-source platforms such as OpenCellID, a database that provides detailed information on each component, including radio type, geographic coordinates, and estimated coverage area. Figure S7 shows the spatial distribution of towers and antennas in Cayey, illustrating how most of the communication components are along the municipality's main roads. The colors in the figure classify the components by radio type.

Figure S7. The communication network component’s location in Cayey, PR.

5. **Accessibility Baseline**

Travel distances to the nearest operational service facilities were calculated using the Dijkstra algorithm and the road network to quantify the accessibility before a hazard event. This initial assessment assumes full functionality before the hazard event. Figure S8 presents accessibility maps and cumulative distribution plots for three essential service facilities: veterinary clinics, eldercare facilities, and schools.

The accessibility maps have a color gradient from light to dark blue, with darker tones representing greater distances from service facilities. Travel distances to the nearest facility range from 0 to 20 km, highlighting spatial disparities in service access. The cumulative distribution plot (Figure S8(d)) shows that approximately 50% of the population resides more than 4 km from these services, with rural areas facing significantly longer travel distances compared to urban areas.

Figure S8. Accessibility maps and the cumulative distribution of travel distances for three essential service facilities pre-hazard: veterinary clinics, eldercare facilities, and schools in Cayey, PR.

6. **Loss of Accessibility to Service Facilities**

***6.1 Hospitals***

A paired t-test was performed to assess changes in building-level distance to operational hospitals before and after the hazard, under the scenario with and without available backup service systems. For the scenario with no backup, the results indicated a statistically significant increase in distance, $(t\left( 21146 \right)=435.58, p<0.001)$ with a mean increase of 12,912.15 meters (95% CI: [12,854.05, 12,970.26]). For the scenario with available power backup in the main hospital, results also indicated a statistically significant increase in distance, $(t\left( 21146 \right)=138.11, p<0.001)$, with a mean distance increase of 1,147.53 meters (95% CI: [1,131.25, 1,163.82]). Although significantly smaller than the scenario without backup systems, this increase still indicates a measurable loss of accessibility to essential services following the hazard event.

***6.2 Grocery Stores***

This section presents the EITD results for grocery store accessibility in the municipality of Cayey. The municipality contains three grocery stores, all located within the urban area. For this analysis, we evaluated a scenario that assumes none of the grocery stores have access to backup power generation. In this scenario, when no grocery store is operational within Cayey during a given simulation iteration, the model assumes residents travel to the nearest operational grocery store outside the municipality. For demonstration purposes, the alternative facilities are located in the neighboring municipalities of Caguas, Cidra, and Aibonito, representing the scenario when multiple neighboring external facilities remain operational.

Figure S9(a) represents the spatial distribution of EITD at the building level for the post-hazard scenario without backup power. Darker shades indicate a greater increase in travel distance to an operational grocery store, representing higher burdens on the affected population. Grocery store facilities that were non-operational in more than 50% of simulation runs are highlighted in red, while those assumed to remain operational due to backup systems are shown in green. This scenario represents the minimum additional travel distance for residents, assuming external grocery stores remain operational. If those external facilities are also closed, actual travel distances would be greater.

Simulation results indicate that in over 75% of iterations, none of the grocery stores in Cayey are operational without backup power, forcing residents to access this service outside the municipality. Consequently, additional travel distances range from no additional distance to over 10 km, particularly in the southern sectors of the municipality.

Figure S9(b) presents the cumulative distribution of travel distances to the nearest operational grocery store under pre- and post-hazard conditions. Under pre-hazard conditions (blue curve), approximately 97% of residents are within 10 km of a grocery store. In contrast, under the post-hazard scenario with no local power backup (red curve), accessibility is significantly reduced, with only 67% of the population remaining within 10 km of an operational grocery store, again assuming external stores are accessible. If they are closed, this travel distance is greater than the one shown in the cumulative distribution.

Figure S9. (a) Spatial distribution of the \ac{EITD} to grocery stores at the building level under a scenario where none of the grocery stores in the municipality has access to backup power generation. (b) Cumulative distribution of travel distance to the nearest operational grocery store for pre-hazard baseline conditions, and when no backup power is available at the grocery stores.

A paired t-test was performed to assess changes in building-level distance to operational grocery store before and after the hazard, under the scenario without available backup service systems. For the scenario with no backup, the results indicated a statistically significant increase in distance, $(t\left( 21146 \right)=414.74, p<0.001)$ with a mean increase of 6,070.49 meters (95% CI: [6,041.80, 6,099.18]).

7. **Community Leaders Interview Questions**

To ensure that the AccES framework integrates local stakeholder knowledge, semi-structured interviews were conducted with community leaders from both rural and urban areas of the case study region. These individuals were identified in collaboration with local governments as key representatives with long-standing involvement.

While the interviews followed a semi-structured format, allowing flexibility for participants to guide the conversation, the following set of questions served as the basis for all interviews. In particular, the first six questions were asked consistently across all participants to identify essential services and access modes. The remaining questions were included in the interviews based on the direction of each conversation to obtain additional insights on hazard impacts, service reliability, and community resilience.

***7.1 Core Questions (asked in all interviews):***

- Briefly describe your community.
- What services are essential to your community’s well-being and functioning?
- Do you believe these essential services are accessible to most community members? Why or why not?
- How do individuals in your community typically access these essential services?
- In what ways do disruptions to essential services affect daily life in your community?
- Which groups within the community are most affected when essential services are disrupted, and why?

***7.2 Additional Questions (asked depending on the interview direction):***

- How would you describe the reliability of essential services in your community?
- What do you think was the greatest hazard-related challenge your community overcame over the past five years?
- What are the most common hazardous events in your community?
- How do those hazards impact your community's access to essential services?
- Is there anything else you want to add about essential services in your community?
- How would you define resilience?
- Do you think your community is resilient?
- How could your community increase its resilience?

**References**

S. Nirandjan, E. E. Koks, M. Ye, R. Pant, K. C. H. Van Ginkel, J. C. J. H. Aerts,

and P. J. Ward. Physical vulnerability database for critical infrastructure multi-hazard

risk assessments: A systematic review and data collection. Natural Hazards and Earth System Sciences Discussions, 2024. Preprint.

Quanta Technology. Cost-benefit analysis of the deployment of utility infrastructure upgrades and storm hardening programs. Technical report, 2009. Final Report.

S. Bjarnadottir, Y. Li, and M. G. Stewart. Hurricane risk assessment of power distribution

poles considering impacts of a changing climate. Journal of Infrastructure Systems, 19(1):12–24, 2013.

A. Shafieezadeh, U. P. Onyewuchi, M. M. Begovic, and R. DesRoches. Age-dependent fragility models of utility wood poles in power distribution networks against extreme wind hazards. IEEE Transactions on Power Delivery, 29(1):131–139, 2014.

Y. E. Teoh, A. Alipour, and A. Cancelli. Probabilistic performance assessment of power

distribution infrastructure under wind events. Engineering Structures, 197:109199,

2019.

Y. Li and B. R. Ellingwood. Hurricane damage to residential construction in the US: Importance of uncertainty modeling in risk assessment. Engineering Structures,

28(7):1009–1018, 2006.

D. Cerrai, P. Watson, and E. Anagnostou. Assessing the effects of a vegetation management standard on distribution grid outage rates. Electric Power Systems Research,

175:105909, 2019.

L. Tian, X. Zhang, and X. Fu. Collapse simulations of communication tower subjected to wind loads using dynamic explicit method. Journal of Performance of Constructed

Facilities, 34(3):04020024, 2020.

D. V. Bilionis and D. Vamvatsikos. Wind performance assessment of telecommunication

towers: A case study in Greece. In COMPDYN Proceedings, pages 5741–5755,

2019.

M. Haklay and P.Weber. OpenStreetMap: User-Generated Street Maps. IEEE Pervasive

Computing, 7(4):12–18, 2008.

Autoridad de Acueductos y Alcantarillados (AAA). Infraestructura de acueductos y sanitaria, 2015. Dataset provided by the Gobierno de Puerto Rico.
